# Calamitean Cones and Their In Situ Spores from the Pennsylvanian Limnic Basins of the Czech Republic

**DOI:** 10.3390/life14060701

**Published:** 2024-05-29

**Authors:** Jiří Bek, Jana Votočková Frojdová

**Affiliations:** Institute of Geology, Academy of Sciences of the Czech Republic, Rozvojová 236, 16500 Prague, Czech Republic; frojdova@gli.cas.cz

**Keywords:** sphenophytes, calamites, in situ spores, *Calamospora*, Pennsylvanian

## Abstract

This paper describes the in situ spores of the *Calamospora* type, macerated from sixty-one specimens of calamitean cones belonging to sixteen species of genera, such as the *Palaeostachya*, *Macrostachya*, *Calamostachys*, and *Huttonia* from the Pennsylvanian Czech Republic period, specifically from the Moscovian/Kasimovian ages (i.e., Duckmantian-Stephanian). The in situ spores were compared to twenty dispersed species of *Calamospora.* The majority of spores were microspores; however, some cones yielded both micro- and megaspores. Morphological variations of the in situ spores, including the diameter, labrum, contact area, ontogenetic stages, and secondary folds of the exine, are described, including their importance for the classification of calamospores. The relationships of *Elaterites*, *Pteroretis*, *Vestispora*, and some monopseudosaccate spores are discussed. All Paleozoic *Calamospora*-producing parent plants are summarized.

## 1. Introduction

Sphenophytes are an important group of both extant and extinct plants. The first specimens come from the Devonian period [1,2,3] but were never abundant. The “Golden age” for sphenophytes was the Carboniferous period, especially in Pennsylvanian times when arborescent forms reached up to 30 m [4]. After the Permian period, their diversity gradually declined until recent times. We know of only one recent genus, *Equisetum* Linnaeus, which has fifteen to eighteen species [4]. Some phylogenetic analyses [5] suggest that sphenophytes and ferns can be a monophyletic group [6]. Paleozoic sphenophytes are divided into two main groups: calamiteans and sphenophyllaleans. All Paleozoic calamiteans produced spores of the *Calamospora* type. Sphenophyllaleans can be palynologically divided into 6–7 groups, and only one of them produced *Calamospora* microspores [7].

*Calamospora* Schopf et al. is a spore genus consisting of mio- and megaspore species with long stratigraphical ranges from the Silurian [8] to Tertiary periods [9]. It is the morphologically simplest spore type, with circular to oval amb, simple trilete rays, and laevigate exine. Many morphologically simple spore types were produced by parent plants of different affinities, including *Calamospora* [7]. This is the reason why *Calamospora* has no stratigraphical and palaeoecological significance.

The genus was established [8] with the species type *C. hartungiana* Schopf et al. The number of classification criteria is low [10,11]. These include the contact area, length of rays of the trilete mark, labrum, number, size, shape, position of the secondary folds of the exine, and diameter. Biologically, calamospores were both male and female spores that cannot be distinguished morphologically but only by their diameter; 200 µm is an artificial “boundary” for the recognition of calamospores into male and female spores [7]. However, this boundary size does not always correspond with the biological function of calamospores. The main producers of *Calamospora* calamitean cones were the genera *Calamostachys* Schimper, *Palaeostachya* Weiss, *Macrostachya* Schimper, and *Paracalamostachys* Weiss. Other parent plants mainly include noeggerathialeans and certain sphenophyllaleans [7]. The relationship of *Calamospora* from *Elaterites* Wilson, *Vestispora* (Wilson & Hoffmeister) Wilson & Venkatachala, and some monopseudosaccate forms of the *Remysporites*/*Auroraspora*/*Perotrilites* type has been discussed [10,12]. This paper describes sixty-one specimens of four calamitean *Calamospora*-producing genera from the Kladno-Rakovník, Radnice, and the Czech part of the Intra-Sudetic basins of the Czech Republic. All palynological aspects of *Calamospora* are summarized, including the morphology, ontogeny, and origin. Their classification is taken from [13]. The most important papers on Carboniferous calamites were published by [13,14], which documented in situ spores [15].

## 2. Geological Setting

**Figure 1:**
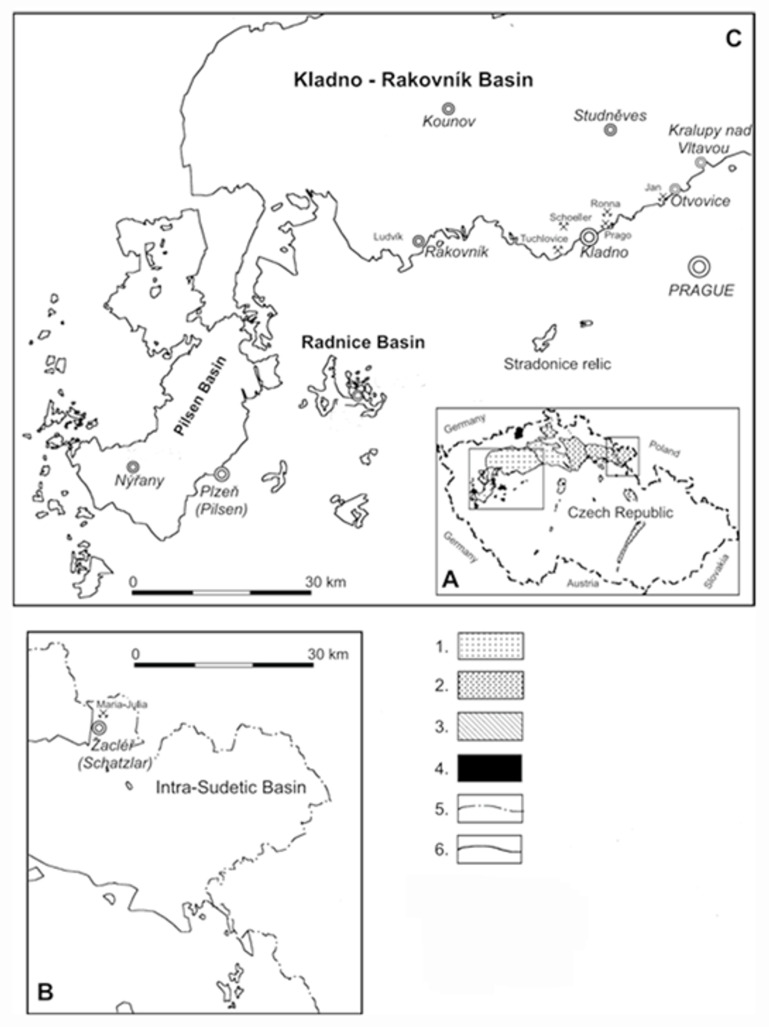
(**A**) Late Palaeozoic continental basins of the Czech Republic. (**B**) Position of localities in the Intra-Sudetic Basin. (**C**) Localities in the basins in central and western Bohemia. Explanation: 1. Central and Western Bohemian Upper Palaeozoic Basins; 2. Lusatian Upper Palaeozoic Basins; 3. Grabens; 4. Krušné Hory Upper Palaeozoic; 5. state border; and 6. present-day limit of continental basins.

Specimens of calamitean cones come from the Kladno-Rakovník Basin, Moscovian-Kasimovian (Upper Duckmantian-Lower Stephanian), Moscovian (Upper Duckmantian) of the Radnice Basin (Figure 1) and Bashkirian-Moscovian of the Intra-Sudetic Basin (Langsettian-Duckmantian, Stephanian, Figure 1).

The Central and Western Permo-Carboniferous limnic basins of the Czech Republic consist of the Kladno-Rakovník, Pilsen, and Radnice basins and a part of the Žihle Basin. The stratigraphical range of these sediments is Moscovian to Kasimovian (Duckmantian to Stephanian) and consists of the Kladno, Týnec, Slaný, and Líně formations. Sediments are up to 1400 m thick [16].

The oldest sediments of the Kladno-Rakovník Basin are early Moscovian (Duckmantian), and the youngest are Gzhelian (upper Stephanian) in age (Table 1).

The Radnice Basin is a small erosional remnant west of the Pilsen Basin (Figure 1). Sedimentation started in the early Moscovian (late Duckmantian) and lasted until the early Permian (Asselian). Stratigraphically, the oldest sediments of the Radnice Member consist of the Radnice group of coals with lower and upper Radnice coal seams and the fossiliferous Whetstone Horizon between them. The Whetstone Horizon is well-known for its rich plant fossils, including the specimens described herein. The lower part of the overlying Nýřany Member is preserved following post-Permian erosion [16,17]. The thickness of members is variable due to the fluvial valleys incised by rivers into the basement.

The Intra-Sudetic Basin belongs to a large complex of Late Palaeozoic continental basins of the Bohemian Massif (Figure 1). The majority of the area (1800 km^2^) is in Poland, with only about a third in the Czech Republic (Figure 1). The sedimentation started in Serpukhovian (late Viséan) and, including several hiatuses, continued for over 80 Ma until the Triassic [18,19]. A detailed overview of the depositional history is described by [17,19]. Serpukhovian (Late Viséan to early Namurian) alluvial deposits of the Blazkow Formation are the oldest in the Czech part of the basin [19]. After a short break, sedimentation resumed in the Bashkirian (late Namurian-middle Westphalian) times by fluvial facies of the Žacléř Formation. After another short break [18], red beds of the Moscovian (late Westphalian to middle Stephanian) Odolov Formation were deposited. The overlying Chvaleč Formation, with other red bed complexes, spans the late Gzhelian (Stephanian) and early Permian (Asselian) periods. The profile continues with the early Permian fluvial red bed [18].

## 3. Material and Methods

The specimens labeled E are housed in the National Museum, Prague, Czech Republic. Some specimens are from the collection of S. Opluštil, Faculty of Sciences, Prague, Czech Republic. A Nikon Eclipse BX51 light microscope was used for the study of palynological slides. Digital photomicrographs of all the in situ microspores are stored in the Laboratory of Palaeobiology and Palaeoecology, Institute of Geology v.v.i., Academy of Sciences, Prague. Spores were recovered by dissolving small portions of sporangia with the aid of nitric acid for 24–40 h and in KOH for 1 h. The majority of spores were mounted in glycerine jelly for a direct microscopic examination. A minority of them were coated with gold and prepared for SEM observation. The terms used for the descriptions of the in situ spores were obtained from the latest edition of the Glossary of Pollen and Spore Terminology [20]. In situ, the spores were compared according to the system of classification of dispersed spores [21,22,23].

## 4. Systematic Paleontology

Equisetales

Calamitaceae

*Palaeostachya* Weiss 1876

Type species: *Palaeostachys elongata* (Presl) Weiss 1876

*Palaeostachya ettingshausenii* Kidston 1903

**Figure 2:**
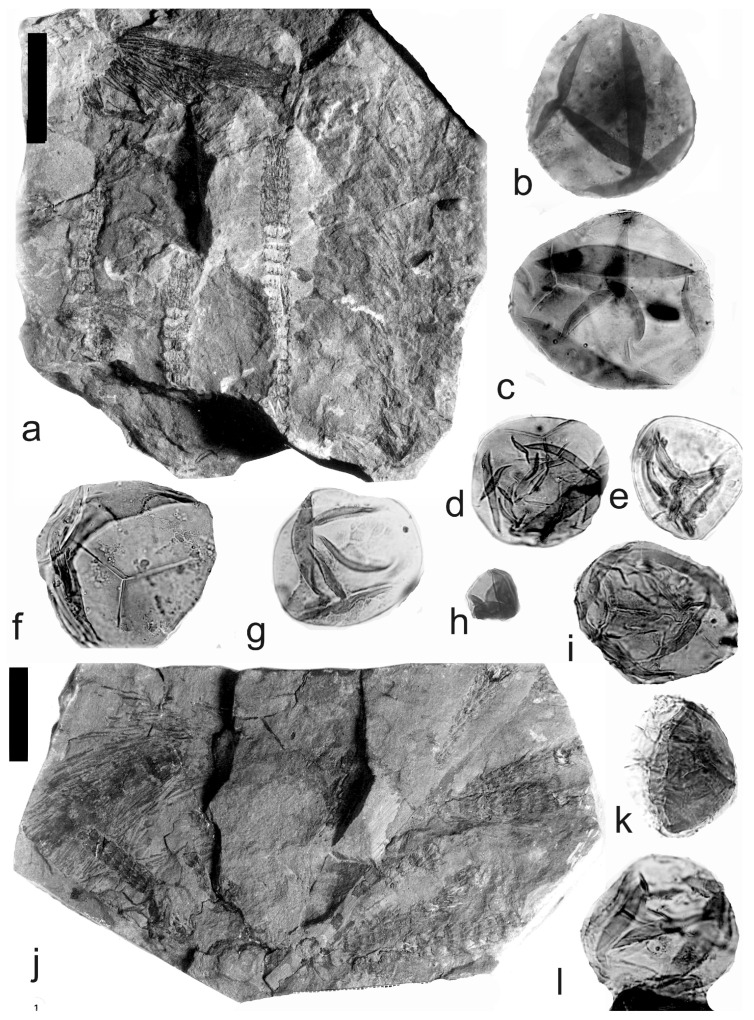
*Palaeostachya ettingshausenii* Kidston. (**a**–**h**) Specimen No. E3622. Maria Julia Mine near Třemošín, Intra-Sudetic Basin, Stephanian. (**a**) General view. Scale bar 20 mm. (**b**,**c**) In situ megaspores of the *Calamospora* type. All ×100. (**d**,**e**) In situ microspores of the *Calamospora* type. Note the number of irregular folds of exine. All ×500. (**f**) In situ microspore of the *Calamospora* type. Note that the rays of the trilete mark reach minimally three-quarters of the radius. ×500. (**g**) In situ microspore of the *Calamospora* type. Note that the rays of the trilete mark reach about three-quarters of the radius. ×500. (**h**) Immature microspore. ×500. (**i**) In situ microspore of the *Calamospora* type. ×500. (**j**) General view of specimen No. E2414. Mayray Mine near Kladno, Kladno-Rakovník Basin, Moscovian. Scale bar 20 mm. (**k**,**l**) In situ microspores of the *Calamospora* type. ×500.

**Figure 3:**
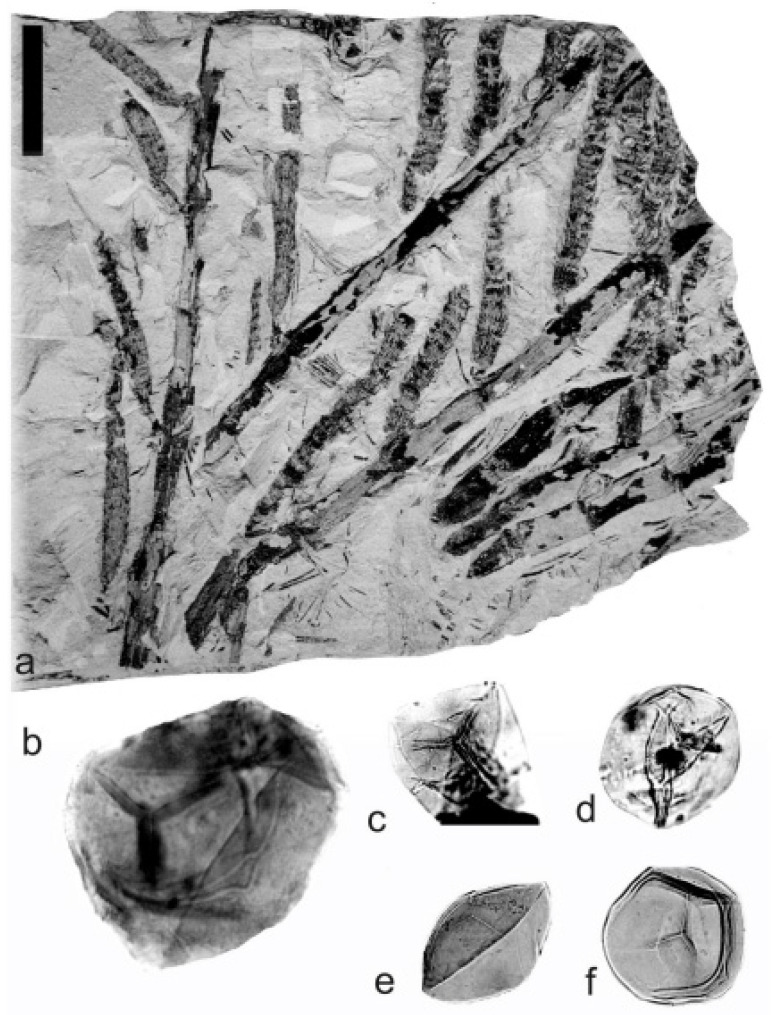
*Palaeostachya ettingshausenii* Kidston. (**a**) General view of the whole specimen No. E3624, Mayray Mine near Kladno, Kladno-Rakovník Basin, Moscovian. Natural size. (**b**) Megaspore of the *Calamopora* type. ×100. (**c**–**f**) Microspores of the *Calamospora* type. (**e**) Note one major fold of exine covers the spore body. (**f**) Note a secondary fold of exine parallels the margin of the spore. All ×500.

**Figure 4:**
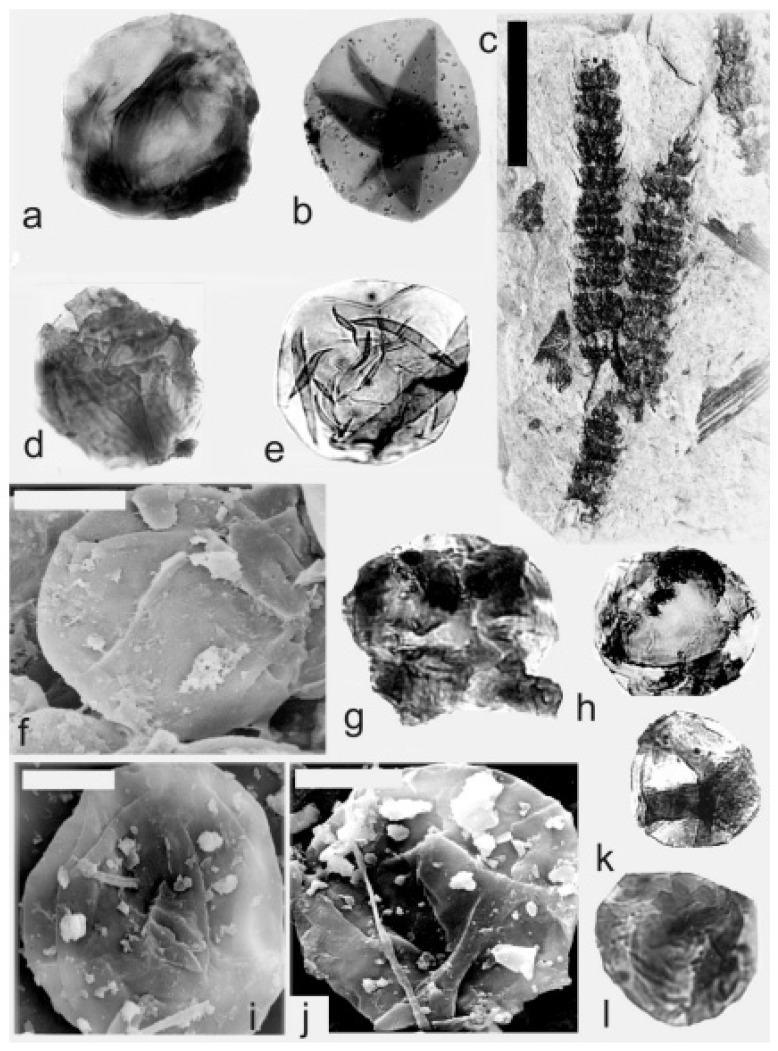
*Palaeostachya ettingshausenii* Kidston. (**a**,**b**) In situ megaspores of the *Calamospora* type isolated from specimen No. 3624, Mayray Mine near Kladno, Kladno-Rakovník Basin, Moscovian. (**c**) Specimen No. E935, Tuchlovice Mine near Kladno, Kladno-Rakovník Basin. Scale bar 175 mm. (**d**,**g**,**h**,**k**,**l**) In situ microspores of the *Calamospora* type. Note that irregular fragments of probable tapetal tissues cover the microspore body. All ×500. (**e**) In situ microspore of the *Calamospora* type. ×500. (**f**,**i**,**j**) In situ microspores of the *Calamospora* type. SEM. Scale bars 20 µm.

Figure 2, Figure 3 and Figure 4

Material: The specimens are from the S. Opluštil’s collection and are as follows: Nos. 935 and 1451 are from the Tuchlovice Mine, Kladno-Rakovník Basin, Czech Republic, Bashkirian (Upper Duckmantian); E2414, E3624, E3626, E2412, and E3623 are from the Mayray Mine, Kladno; E3618 is from the Max Mine, Kladno; E3635 is from the Ronna Mine, Kladno; and E3596 is from the Břasy locality. All are from the Kladno-Rakovník Basin. The following specimens are from the Intra-Sudetic Basin as follows: E3622 is from the Maria-Julia Mine near Třemošín, Kasimovian (Stephanian); and E2500 is from the Žacléř locality, with an unknown stratigraphic position.

A description of the in situ spores is as follows: The trilete spores are circular, subtriangular, or oval in amb. Two kinds of spores probably represent the microspores (Figure 2d–i,k,l, Figure 3c–f, and Figure 4c,d,f–i) and megaspores (Figure 2b,c, Figure 3b, and Figure 4a,b). The laevigate exine of the microspores is up to 4 µm thick, and that of the megaspores is 6–9 µm thick. The rays of the trilete mark reach a third to two-thirds of the radius. Secondary folds of the exine possess variable sizes, shapes, positions, and numbers. Three size categories of the in situ spores were recognized (Table 2): the first with a size range of 44 (71) 171 µm (the majority of specimens), and the second is 80 (139) 181 µm; the megaspores are 234 (448) 708 µm. The contact area is sometimes developed. The in situ microspores can be compared to the dispersed miospore species *Calamospora microrugosa* (Ibrahim) Schopf et al., *C.* cf. *microrugosa*, *C. pedata* Kosanke, *C.* cf. *pedata*, *C. pallida* (Loose) Schopf et al., *C.* cf. *pallida*, *C.* cf. *liquida*, *C. breviradiata* Kosanke, and *C. hartungiana* Schopf et al., and the megaspores to the species *C. perrugosa* (Loose) Schopf et al.

*Palaeostachya distachya* (Sternberg) Jongmans 1911

**Figure 5:**
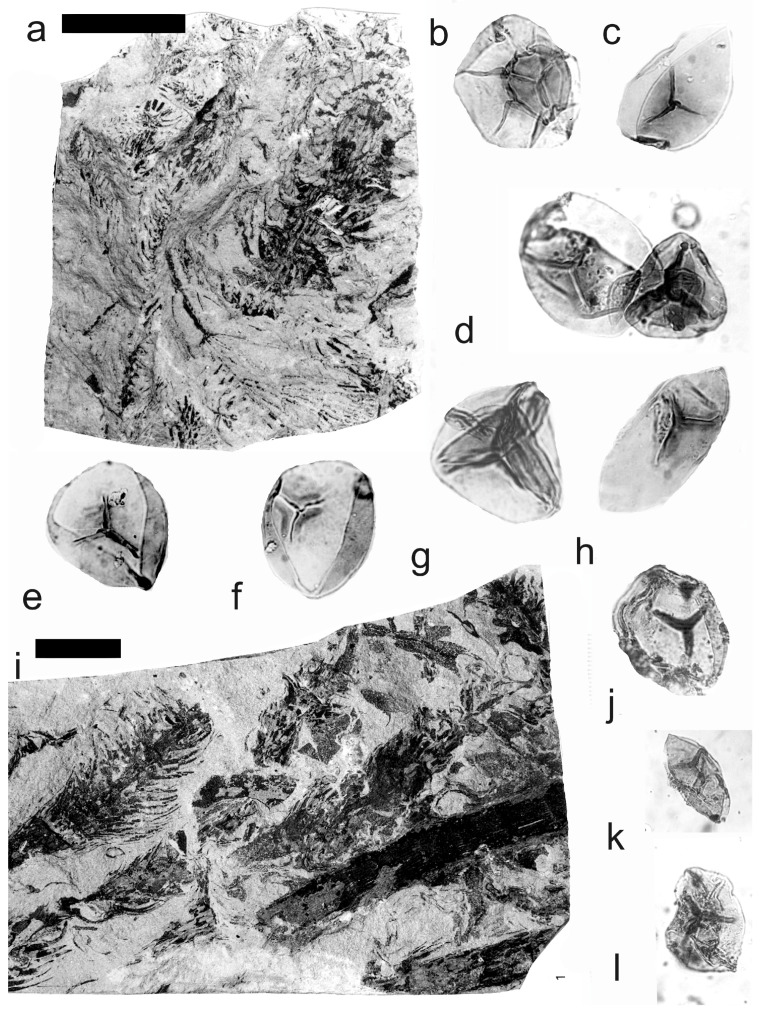
*Palaeostachya distachya* (Sternberg) Jongmans. (**a**) Specimen No. E1132, Ronna Mine near Kladno, Kladno-Rakovník Basin, Moscovian. Scale bar 20 mm. (**b**) In situ microspore of the *Calamospora* type. Note the dark contact area. ×500. (**c**) In situ microspore of the *Calamospora* type. Note that one major fold covers the microspore body. ×500. (**d**) Two in situ microspores of the *Calamospora* type. The left specimen represents a mature microspore, while the right specimen is relatively immature. ×500. (**e**) In situ microspore of the *Calamospora* type. Note the two major folds of exine. ×500. (**f**) In situ microspore of the *Calamospora* type. Note the dark, thickened labrum. ×500. (**g**) In situ microspore of the *Calamospora* type. ×500. (**h**) In situ microspore of the *Calamospora* type. Note the dark labrum. ×500. (**i**) Specimen No. E3608, Hnidousy near Kladno, Kladno-Rakovník Basin, Moscovian. Scale bar 20 mm. (**j**) In situ microspore of the *Calamospora* type. Note the dark, thin labrum. ×500. (**k**,**l**) In situ microspores of the *Calamospora* type. All ×500.

**Figure 6:**
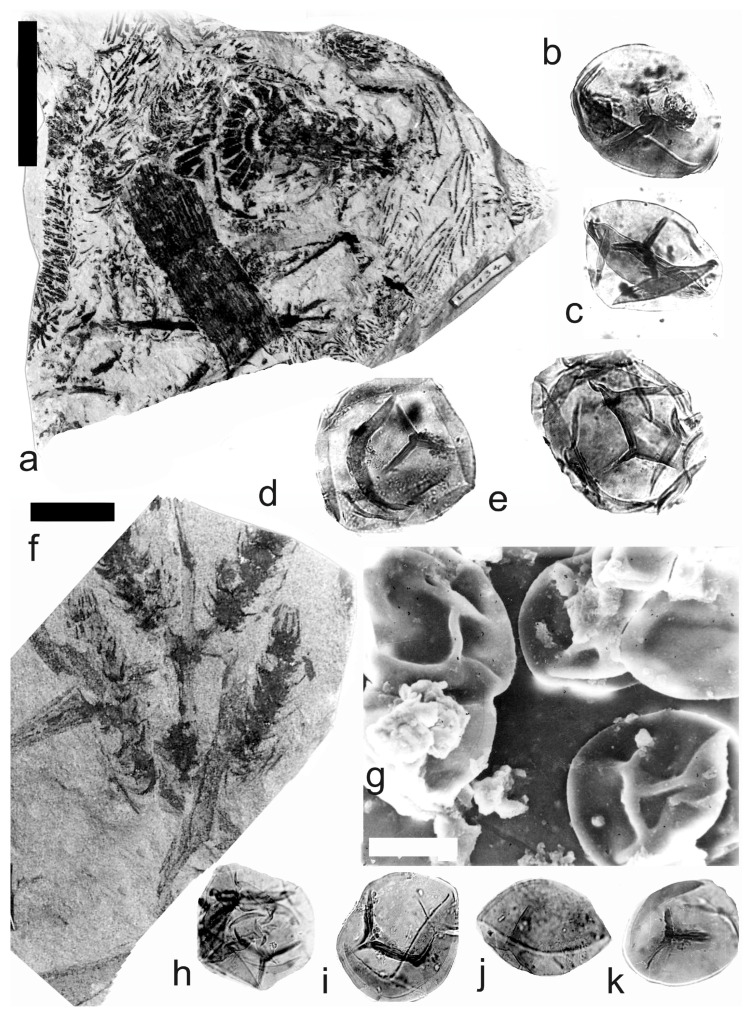
(**a**–**e**) *Palaeostachya distachya* (Sternberg) Jongmans. (**a**) Specimen No. E3608, Hnidousy near Kladno, Kladno-Rakovník Basin, Moscovian. Scale bar 20 mm. (**b**) In situ microspore of the *Calamospora* type. Note that one major fold covers the microspore body. ×500. (**c**) In situ microspore of the *Calamospora* type. Note the dark labrum. ×500. (**d**,**e**) In situ microspores of the *Calamospora* type. All ×500. (**f**–**k**) *Palaeostachya pedunculata* Williamson. (**f**) Specimen No. E1125, Ronna Mine Kladno, Kladno-Rakovník Basin, Moscovian. Scale bar 10 mm. (**g**) In situ microspores of the *Calamospora* type. Note the elevated labrum. SEM. Scale bar 25 µm. (**h**–**k**) In situ microspores of the *Calamospora* type. All ×500.

Figure 5 and Figure 6a–e

Material: E3608, E3598, and E3604 are from Hnidousy, near Kladno; and E1132, E3599, E3603, E1132, and E1134 are from the Ronna Mine. All of them are from the Kladno-Rakovník Basin, Bashkirian-Moscovian (Upper Duckmantian/Lower Bolsovian).

A description of the in situ spores is as follows: The trilete spores are circular, subtriangular, or oval in amb. The laevigate exine of the microspores is up to 3 µm thick. The rays of the trilete mark reach a third to two-thirds of the radius. Secondary folds of the exine possess variable sizes, shapes, positions, and numbers. The size range is 34 (65) 119 µm (Table 3). The contact area (Figure 5b–d) and labrum (Figure 5f,j,l and Figure 6d,e) are sometimes developed. The in situ microspores can be compared to the dispersed miospore species *Calamospora mutabilis* (Loose) Schopf et al., *C.* cf. *pedata*, *C. minuta* Bharadwaj, *C.* cf. *minuta*, *C. hartungiana*, *C.* cf. *hartungiana*, *C. breviradiata*, *C.* cf. *breviradiata*, and *C saariana* Bharadwaj.

*Palaeostachya pedunculata* Williamson 1874

Figure 6f–k

Material: No. E1125, from the Ronna Mine near Kladno, Kladno-Rakovník Basin, Bashkirian (Upper Duckmantian).

A description of the in situ spores is as follows: The trilete spores are circular, subtriangular, or oval in amb. The laevigate exine of the microspores is up to 2 µm thick. The rays of the trilete mark reach half of the radius. Secondary folds of the exine possess variable sizes, shapes, positions, and numbers (Figure 6g–k). The size range is 51 (70) 89 µm. The labrum is sometimes developed (Figure 6g,i). The in situ microspores can be compared to the dispersed miospore species *Calamospora* cf. *pedata*, *C.* cf. *microrugosa*, and *C.* cf. *straminea.*

*Palaeostachya elongata* (Presl) Weiss 1976

Figure 7a–o

Material: No. E1122 is from Bashkirian (Upper Duckmantian), Kladno locality, Kladno-Rakovník Basin; No. E1121 is from Moscovian (Bolsovian), Rakovník locality, Kladno-Rakovník Basin; Nos. E3607 and E 3616 are from Moscovian (Bolsovian), the Šamotka locality, Věnec Coal Seam near Lubná; No. E3631 is from Bashkirian (Langsettian-Duckmantian) of the Intra-Sudetic Basin, Maria-Julia Mine near Žacléř.

**Figure 7:**
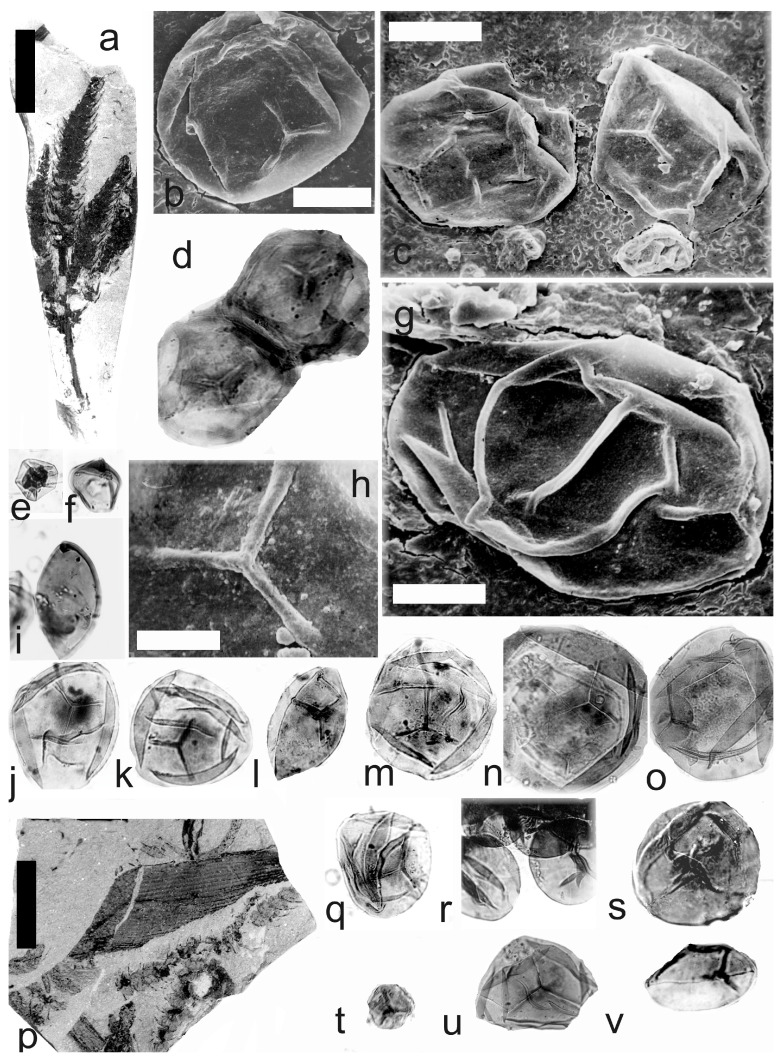
(**a**–**o**) *Palaeostachya elongata* (Presl) Weiss. (**a**) Specimen No. E1122, Kladno Locality, Kladno-Rakovník Basin, Moscovian. Scale bar 20 mm. (**b**,**c**) In situ microspores of the *Calamospora* type. SEM, scale bar 20 µm. (**d**) Two in situ microspores of the *Calamospora* type with fragments probably of tapetal tissues. ×500. (**e**,**f**) Two immature in situ microspores. All ×500. (**g**) In situ microspore of the *Calamospora* type. SEM, scale bar 20 µm. (**h**) Elevated labrum of in situ microspore of the *Calamospora* type. Detail of Figure (**c**) (right specimen). SEM, scale bar 10 µm. (**i**) In situ microspore of the *Calamospora* type. Note one major fold covering the body. ×500. (**j**,**k**,**m**–**o**) In situ microspores of the *Calamospora* type. Note the dark contact area. All ×500. (**l**) In situ microspore of the *Calamospora* type. Note one major fold covers half of the spore body. ×500. (**p**–**v**) *Palaeostachya gracillima* Weiss. (**p**) Specimen No. E1119. Kladno locality, Kladno-Rakovník Basin. Moscovian. Scale bar 20 mm. (**q**–**s**,**u**) In situ microspores of the *Calamospora* type. All ×500. (**t**) Immature in situ microspore. ×500. (**v**) In situ microspore of the *Calamospora* type. Note one major fold of the exine covers half of the spore body. ×500.

A description of the in situ spores is as follows: The trilete spores are circular, subtriangular, or oval in amb. The laevigate exine of the microspores is up to 2 µm thick. The rays of the trilete mark reach a third to two-thirds of the radius. Secondary folds of the exine possess variable sizes, shapes, positions, and numbers (Figure 7b–d,j–o). The size range is 60 (112) 141 µm (Table 4). The contact area and labrum are up to 6 µm in size and sometimes developed (Figure 7c,h,j,l,n,o). Sometimes, fragments of the perispore can occur on the surface of the exine (Figure 7d). The in situ microspores can be compared to the dispersed miospore species *Calamospora hartungiana*, *C.* cf. *hartungiana*, *C. falkenbergensis* Venkatachala & Bharadwaj, and *C. mutabilis*. It is possible that some spores that are classified as *Calamospora falkenbergensis*, *C.* cf. *hartungiana,* and *C. mutabilis*, with size ranges of 82 to 152 µm, may be megaspores.

**Figure 8:**
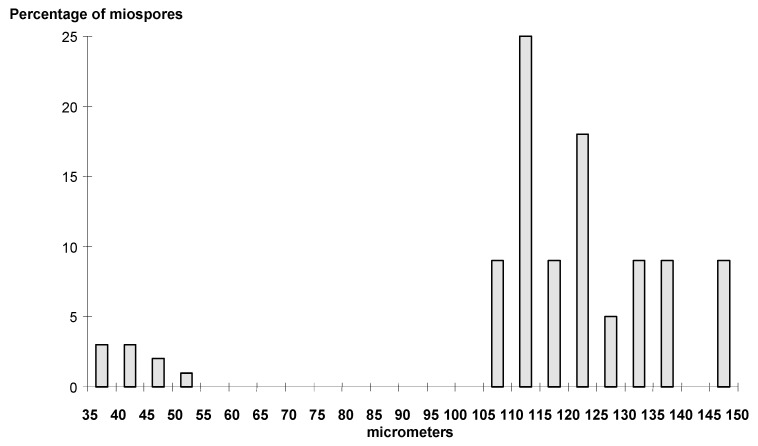
Size distribution of the in situ spores of *Palaeostachya elongate* (Presl) Weiss, (E 3631).

Figure 8 shows the bimodal distribution of the in situ spores isolated from *Palaeostachya elongata*.

*Palaeostachya gracillima* Weiss 1876

Figure 7p–v

Material: No. E1119 is from the Kladno locality, Kladno-Rakovník Basin, Moscovian (Bolsovian), and No. E1127 is from the Příčina-Na Brantech locality near Lubná, Kladno-Rakovník Basin, Moscovian (Bolsovian).

A description of the in situ spores is as follows: Trilete spores are circular, subtriangular, or oval in amb. The laevigate exine of the microspores is up to 2 µm thick. The rays of the trilete mark reach a third to two-thirds of the radius. Secondary folds of the exine possess variable sizes, shapes, positions, and numbers (Figure 7q–v). The size range is 34 (65) 95 µm (Table 5). The labrum is 2–4 µm broad and is sometimes developed (Figure 7v). The in situ microspores can be compared to the dispersed miospore species *Calamospora braviradiata*, *C. minuta*, *C.* cf. *pedata*, and *C.* cf. *microrugosa.*

*Palaeostachya feistmantelii* Němejc 1953

Figure 9a–f

Material: Nos. E3492 and E3493 are from the Štilec locality near Žebrák, Kladno, Rakovník Basin, Moscovian (Bolsovian).

**Figure 9:**
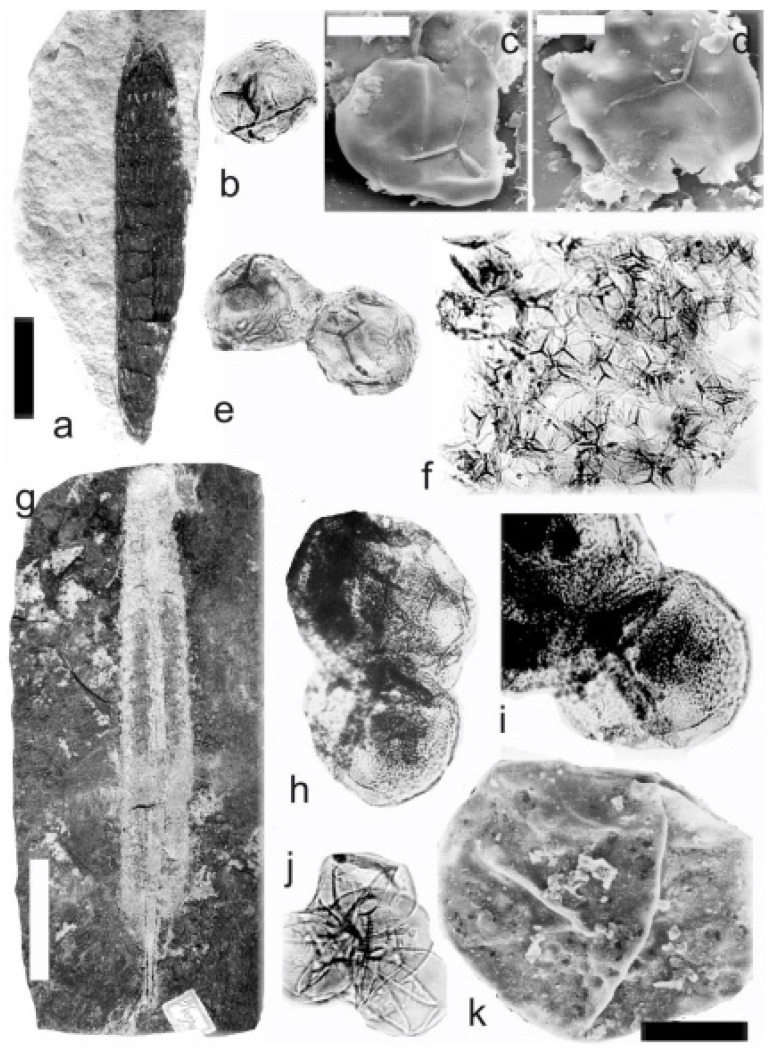
(**a**–**f**) *Palaeostachya feistmantelii* Němejc. (**a**) Specimen No. E3492. Štilec locality near Žebrák, Kladno-Rakovník Basin. Moscovian. Scale bar 20 mm. (**b**,**e**) In situ microspores of the *Calamospora* type. All ×500. (**c**,**d**) Fragments of in situ microspores of the *Calamospora* type. SEM, scale bar 20 µm. (**f**) Mass of damaged in situ microspores of the *Calamospora* type. ×500. (**g**–**k**) *Macrostachya carinata* (Germar) Zeiller. (**g**) Specimen No. E3637, Mirošov locality, Kladno-Rakovník Basin, Moscovian. Scale bar 50 mm. (**h**,**i**) In situ megaspores of the *Calamospora* type. All ×150. (**j**) In situ microspores of the *Calamospora* type. ×500. (**k**) In situ megaspore of the *Calamospora* type. SEM, scale bar 80 µm. *Macrostachya* Schimper 1869.

A description of the in situ spores is as follows: The trilete are subcircular microspores. The size range is 55 (69) 108 µm. The laevigate exine is up to 2 µm thick. The labrum is 2–4 µm in size (Figure 9b,e). Rays of the trilete mark reach a third to two-thirds of the radius. The microspores are poorly preserved and only as fragments and incomplete specimens (Figure 9c,d,f), probably due to oxidation of the rock. This is the reason why in situ spores can be only described as the *Calamospora* type.

**Figure 10:**
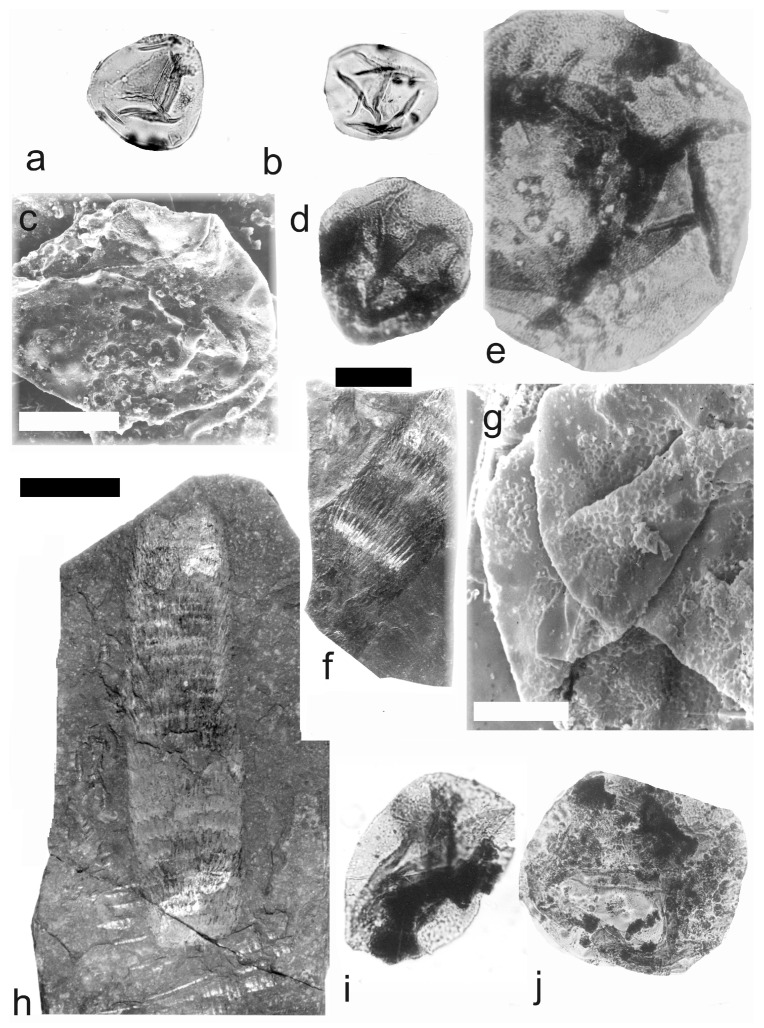
*Macrostachya carinata* (Germar) Zeiller. (**a**–**e**) Specimen No. E3637, Mirošov locality, Kladno-Rakovník Basin, Moscovian. (**a**,**b**) In situ microspores of the *Calamospora* type. All ×500. (**c**) In situ megaspore of the *Calamospora* type. SEM, scale bar 170 µm. (**d**,**e**) In situ megaspores of the *Calamospora* type. (**d**) ×120. Scale bar, (**e**) ×250. (**f**,**g**) Specimen No. E1180, Mirošov locality, Kladno-Rakovník Basin, Moscovian. (**f**) General view of the specimen. Scale bar 20 mm. (**g**) In situ megaspore of the *Calamospora* type. SEM. Note the negative sculpture of exine. Scale bar 700 µm. (**h**–**j**) Specimen No. E1181, Mirošov locality, Kladno-Rakovník Basin, Moscovian. (**h**) General view of the specimen. Scale bar 20 mm. (**i**,**j**) In situ megaspores of the *Calamospora* type. (**i**) ×120, (**j**) ×200.

**Figure 11:**
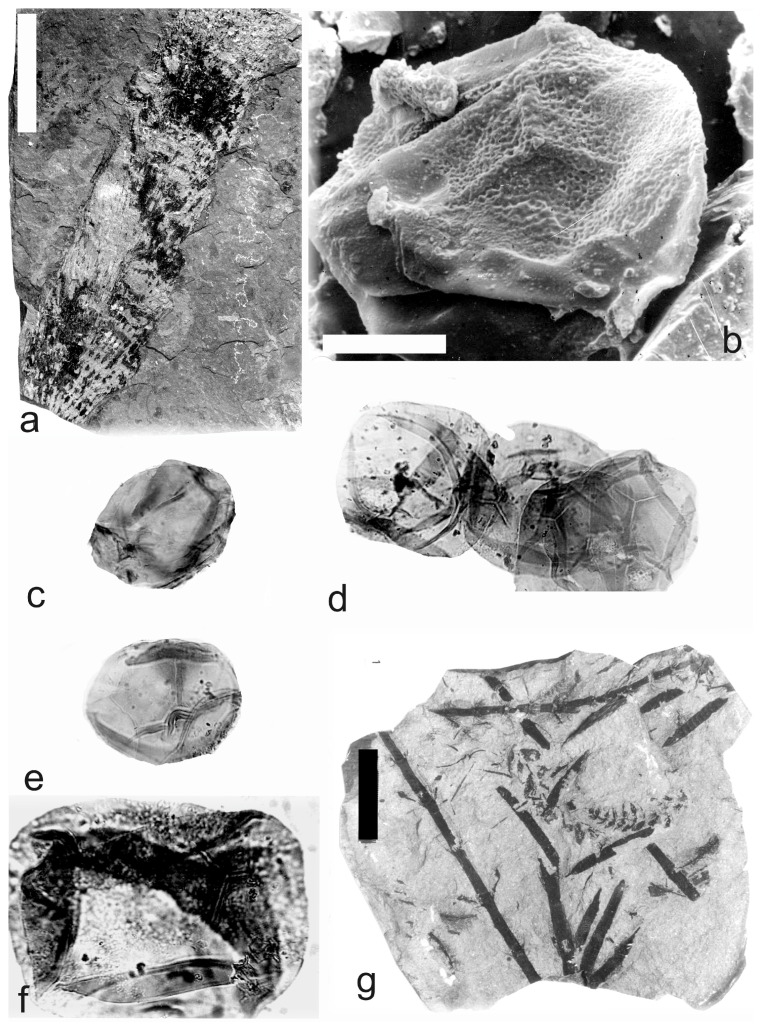
(**a**–**f**) *Macrostachya carinata* (Germar) Zeiller. (**a**) Specimen No E1178, Mirošov locality, Kladno-Rakovník Basin, Moscovian. Scale bar 40 mm. (**b**) In situ megaspore of the *Calamospora* type. Note the slightly elevated contact area and elevated labrum. SEM, scale bar 70 µm. (**c**–**e**) In situ microspores of the *Calamospora* type. All ×500. (**f**) In situ megaspore of the *Calamospora* type. ×150. (**g**) *Calamostachys germanica* Weiss, specimen E5641, Na Brantech locality, Lubná, Kladno-Rakovník Basin, Moscovian. Scale bar 40 mm.

Figure 9g–k, Figure 10 and Figure 11a–f

*Macrostachya carinata* (Germar) Zeiller 1879

Material: Collection of S. Opluštil, No. 861 from the Slaný Mine, Kladno-Rakovník Basin, Moscovian. No. E3641 is from the Mirošov locality, Kladno-Rakovník Basin, Moscovian (Asturian) E1178, E1181 (material type).

A description of the in situ spores is as follows: The trilete spores are circular, subtriangular, or oval in amb. The laevigate exine of the microspores (Figure 9j and Figure 10a,b) is up to 2 µm and the megaspores (Figure 9h,i,k, Figure 10c–e,i,j, and Figure 11b–f) are up to 3 µm thick. The rays of the trilete mark reach a third to three-quarters of the radius. Secondary folds of the exine possess variable sizes, shapes, positions, and numbers (Figure 9j, Figure 10a,b,e, and Figure 11e,d). The size range (Table 6) of the microspores is 54 (76) 125 µm, and the megaspores are 156 (265) 372 µm (Table 7). The contact area and labrum are 2–4 µm in size and sometimes developed. The in situ microspores can be compared to the dispersed miospore species *Calamospora mutabilis*, *C.* cf. *pedata*, and *C.* cf. *liquida*. Megaspores are classified as *Calamospora* sp. due to their large diameters.

*Calamostachys* Schimper 1869

*Calamostachys germanica* Weiss 1976

**Figure 12:**
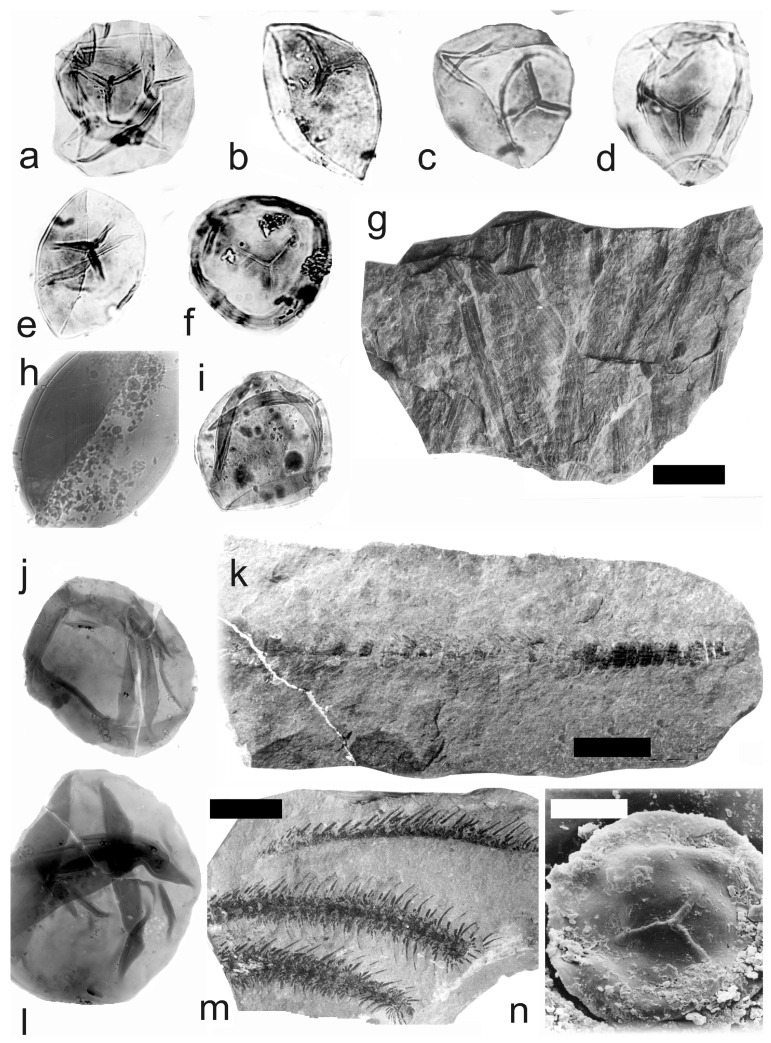
*Calamostachys germanica* Weiss. (**a**–**f**) Specimen E5641, Na Brantech locality, Lubná, Kladno-Rakovník Basin, Moscovian. (**a**–**f**) In situ microspores of the *Calamospora* type. Note the dark contact area (**a**,**c**–**f**). All ×500. (**g**–**j**) Specimen E5641, Třemošná locality, Lubná, Kladno-Rakovník Basin, Moscovian. (**g**) General view of the specimen. Scale bar 20 mm. (**h**,**j**) In situ megaspores of the *Calamospora* type. (**h**) ×150, (**j**) ×90. (**i**) In situ microspore of the *Calamospora* type. ×500. (**k**,**l**) Specimen No. E2409, Třemošná locality, Lubná, Kladno-Rakovník Basin, Moscovian. (**k**) General view of specimen. Scale bar 20 mm. (**l**) In situ megaspore of the *Calamospora* type. ×160. (**m**,**n**) Specimen No. E1159, Třemošná locality, Lubná, Kladno-Rakovník Basin, Moscovian. (**m**) General view of specimen. Scale bar 20 mm. (**n**) In situ microspore of the *Calamospora* type. Proximal surface. SEM, scale bar 20 µm.

**Figure 13:**
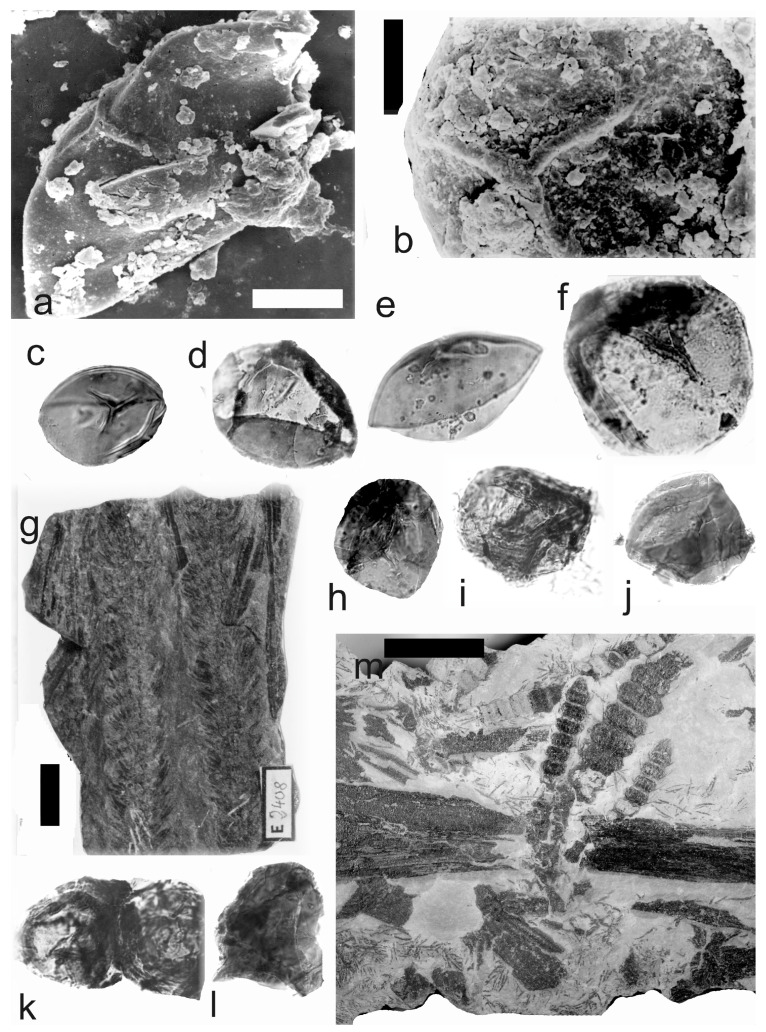
(**a**–**k**) *Calamostachys germanica* Weiss. (**a**–**f**) Specimen No. E1159, Třemošná locality, Lubná, Kladno-Rakovník Basin, Moscovian. Scale bar. (**a**) In situ microspore of the *Calamospora* type. SEM, scale bar 20 µm. (**b**) In situ megaspore of the *Calamospora* type. SEM, scale bar 40 µm. (**c**–**e**) In situ microspores of the *Calamospora* type. ×500. (**f**) In situ megaspore of the *Calamospora* type. Scale bar. (**g**–**l**) Specimen No. E2408, Ignác Mine, Kladno-Rakovník Basin, Moscovian. (**g**) General view of the specimens. Scale bar 15 mm. (**h**–**l**) In situ microspores of the *Calamospora* type. Note the irregular fragments of the exospore. All ×500. (**m**) *Calamostachys incrassata* Němejc. Specimen No. E. E1114, V Krčeláku locality, Rako Mine near Lubná, Kladno-Rakovník Basin, Moscovian. Scale bar 50 mm.

Figure 11g, Figure 12 and Figure 13a–l

Material: Nos. E1161 and E2409 are from the Třemošná locality, Kladno-Rakovník Basin, Moscovian (Bolsovian); No. E2408 is from the Ignác Mine, Kladno-Rakovník Basin, Moscovian (Bolsovian); No. E3620 is from the Kladno locality, Kladno-Rakovník Basin, Moscovian (Bolsovian); No. E5641 is from the Na Brantech locality, Lubná, Kladno-Rakovník Basin, Moscovian (Bolsovian).

A description of the in situ spores is as follows: The trilete spores are circular, subtriangular, or oval in amb. The laevigate exine of the microspores is up to 2 µm thick. The rays of the trilete mark reach a quarter to a half of the radius. Secondary folds of the exine possess variable sizes, shapes, positions, and numbers (Figure 12a–h and Figure 13c–j). The size range of the microspores is 54 (94) 126 µm, and the megaspores are 180 (357) 684 µm (Table 8). The labrum is 2–4 µm broad, and the dark contact areas (Figure 12a,d,f) are sometimes developed (Figure 12e and Figure 13a). The in situ microspores can be compared to the dispersed miospore species *Calamospora microrugosa*, *C.* cf. *mutabilis*, *C. pallida*, *C.* cf. *pallida*, *C.* cf. *pedata*, *C. breviradiata*, and *C. hartungiana*; the megaspores are classified as *Calamospora* sp. due to their large diameters (up to 684 µm).

*Calamostachys incrassata* Němejc 1953

**Figure 14:**
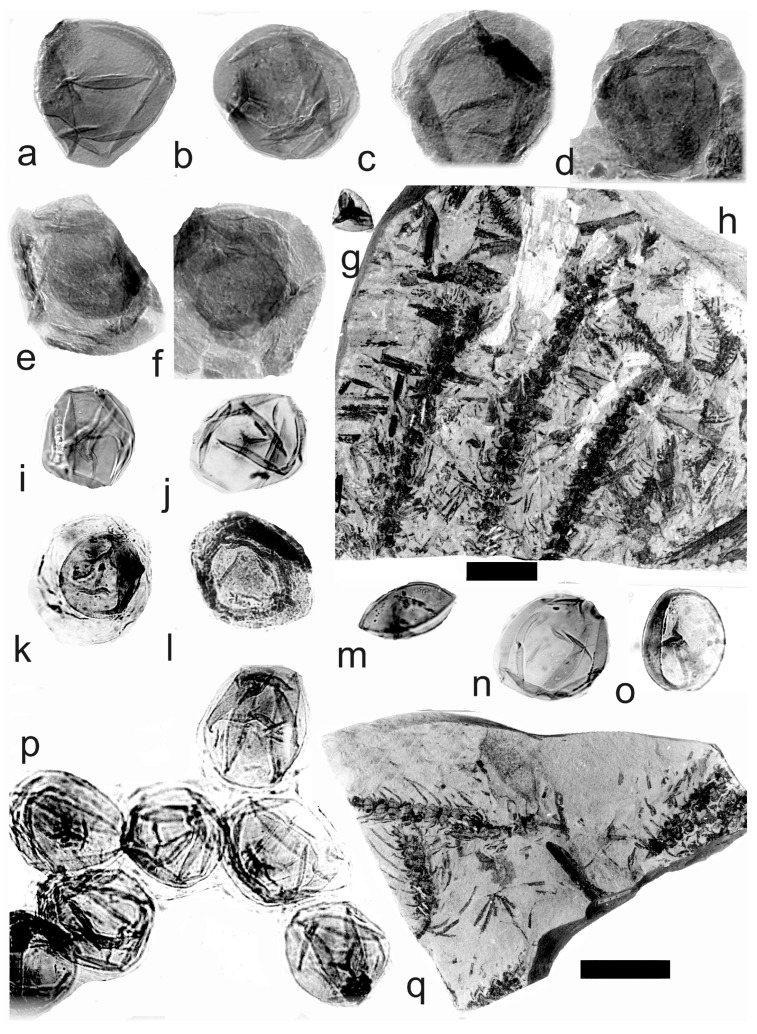
(**a**–**g**) *Calamostachys incrassata* Němejc, specimen No. E. E1114, V Krčeláku locality, Rako Mine near Lubná, Kladno-Rakovník Basin, Moscovian. (**a**–**f**) In situ microspores of the *Calamospora* type. Note the fragments of probably tapetal tissues. All ×500. (**g**) Immature in situ microspore. ×500. (**h**–**q**) *Calamostachys longibracteata* Němejc. (**h**–**p**) Specimen No. E1163, Mayrau Mine, Vinařice, Kladno-Rakovník Basin, Moscovian. (**h**) General view of the specimen. Scale bar 20 mm. (**i**,**j**,**n**,**o**) In situ microspores of the *Calamospora* type. All ×500. (**k**) In situ microspore of the *Calamospora* type. Note that monopseudosaccate-like layer envelopes the inner body of the *Calamospora* type. ×500. (**l**) In situ microspore of the *Calamospora* type. Note the monopseudosaccate-like layer. ×500. (**m**) In situ microspore of the *Calamospora* type. Note that one major fold covers the microspore body. ×500. (**p**) In situ microspores of the *Calamospora* type. Note that monopseudosaccate-like exine layers enveloped central bodies. ×450. (**q**) Specimen No. E1155, Max Mine, Libušín, Kladno-Rakovník Basin, Bolsovian. Scale bar 30 mm.

Figure 13m and Figure 14a–g

Material: No. E1114, is from the V Krčeláku locality, Rako Mine near the Lubná, Kladno-Rakovník Basin, Moscovian (Bolsovian).

A description of the in situ spores is as follows: The trilete spores are circular, subtriangular, or oval in amb. The laevigate exine of the microspores is up to 2 µm thick. The rays of the trilete mark reach a third of the radius. Secondary folds of the exine possess variable sizes, shapes, positions, and numbers (Figure 14a–f). The size range is 51 (73) 88 µm. The labrum is 2–4 µm broad and is sometimes developed. The in situ microspores can be compared to the dispersed miospore species *Calamospora* cf. *hartungiana*. Some spores are 54 (75) 94 µm large and are enveloped in a very thin monopseudosaccate layer (Figure 14c–f) and may resemble some forms of the miospores genera *Auroraspora* Hoffmeister et al., *Remysporites* Butterworth & Williams, *Perotrilites* Couper, *Phyllothecotriletes* Luber, or even *Diaphanospora* Balme.

*Calamostachys longibracteata* Němejc 1953

**Figure 15:**
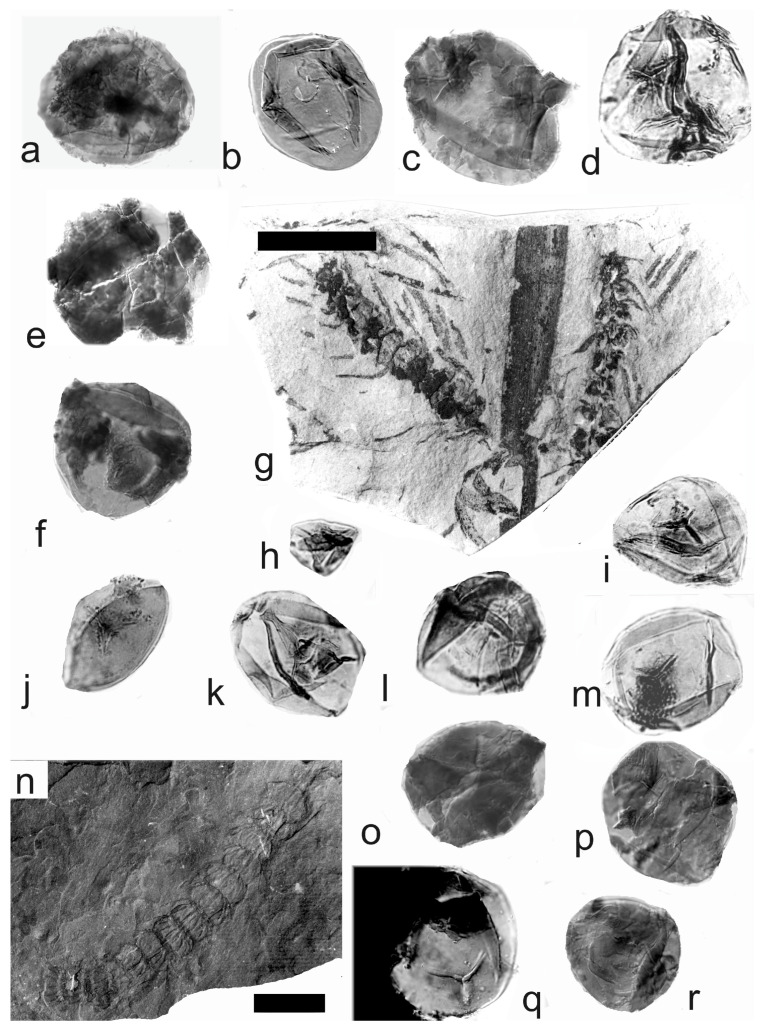
(**a**–**m**) *Calamostachys longibracteata* Němejc. (**a**–**f**) Specimen No. E1155, Max Mine, Libušín, Kladno-Rakovník Basin, Moscovian. (**a**,**c**,**e**,**f**) In situ microspores of the *Calamospora* type. Note the fragments of the monopseudosaccate-like exine layer. All ×500. (**b**,**d**) In situ microspores of the *Calamospora* type. All ×500. (**g**–**m**) Specimen E1154. Max Mine, Libušín, Kladno-Rakovník Basin. Moscovian. General view. (**g**) General view of the specimen. Scale bar 20 mm. (**h**) Immature in situ microspore. ×500. (**i**) In situ microspore of the *Calamospora* type. ×500. (**j**) In situ microspore of the *Calamospora* type. Note that one major fold of exine covers the body. ×500. (**k**–**m**) In situ microspores of the *Calamospora* type. All ×500. (**n**–**r**) *Calamostachys tuberculata* Sternberg, E 1147, Doubrava locality, Kladno-Rakovník Basin, Moscovian. General view. Scale bar. (**n**) General view of specimen. Scale bar 20 mm. (**o**,**p**,**r**) In situ microspore of the *Calamospora* type. Note the fragments of probable tapetal tissues. All ×500. (**q**) In situ microspores of the *Calamospora* type. ×500.

Figure 14h–q and Figure 15a–m

Material: No. E3605 is from the Kladno locality, Kladno-Rakovník Basin, Moscovian (Bolsovian); Nos. E1154 and E1155 are from the Max Mine, Libušín, Kladno-Rakovník Basin, Moscovian (Bolsovian); No. E1163 is from the Mayrau Mine, Vinařice, Kladno-Rakovník Basin, Moscovian (Bolsovian).

A description of the in situ spores is as follows: The trilete spores are circular, subtriangular, or oval in amb. The laevigate exine of the microspores is up to 2 µm thick. The rays of the trilete mark reach a third to two-thirds of the radius. Secondary folds of the exine possess variable sizes, shapes, positions, and numbers (Figure 14a–c,i,j,m–o). The size range is 45 (60) 83 µm (Table 9). Sometimes, the outer perispore-like layer envelopes the central body of the *Calamospora* type (Figure 14k,l,p). The in situ microspores can be compared to the dispersed miospore species *Calamospora microrugosa*, *C. pallida*, and *C. pedata.*

*Calamostachys tuberculata* (Sternberg) Jongmans 1911

**Figure 16:**
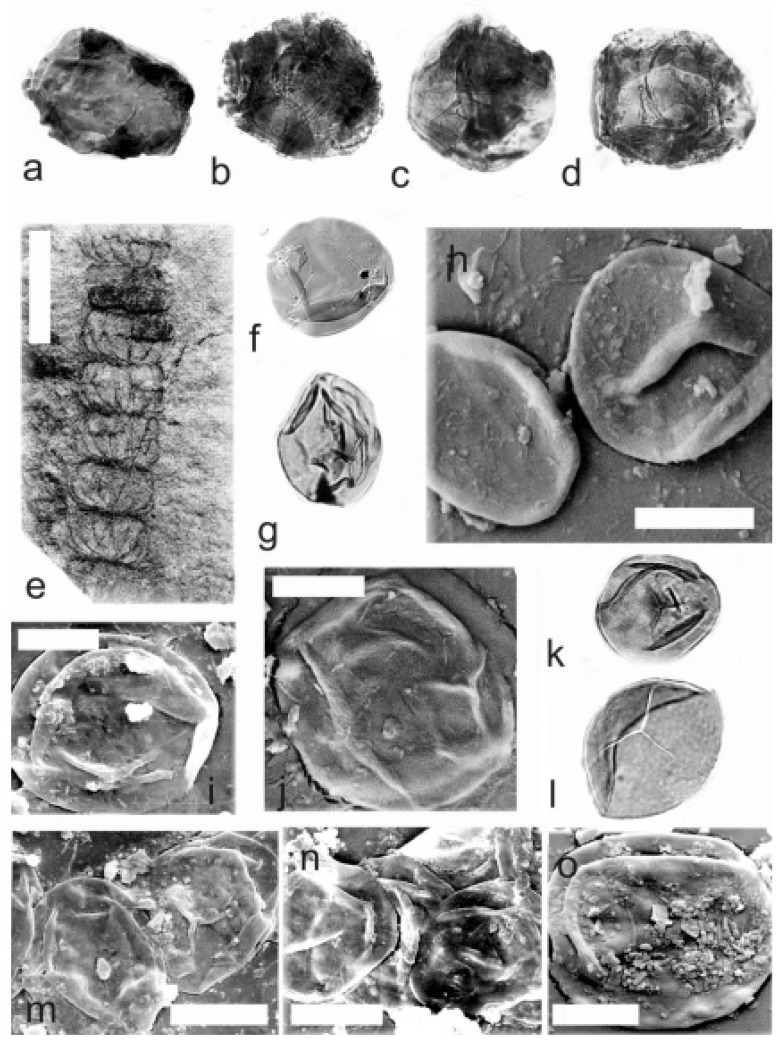
*Calamospora tuberculata* Sternberg. (**a**–**d**) Specimens E 1147, Doubrava locality, Kladno-Rakovník Basin, Moscovian. (**a**–**d**) In situ microspores of the *Calamospora* type. Note the irregular fragments of probable tapetal tissues. All ×500. (**e**–o) Specimen 1951, Kladno locality, Kladno-Rakovník Basin, Kasimovian. (**e**) General view of the specimen. Scale bar 15 mm. (**f**,**g**,**k**,**l**) In situ microspores of the *Calamospora* type. All ×500. (**h**–**j**,**m**–**o**) In situ microspores of the *Calamospora* type. SEM. (**h**) Scale bar 40 µm. (**i**) Scale bar 35 µm. (**j**) Scale bar 30 µm. (**m**) Scale bar 40 µm. (**n**) Scale bar 40 µm. (**o**) Scale bar 25 µm.

Figure 15n–r and Figure 16

Material: Collection of S. Opluštil: Nos. 1238 and 1951 are from the Kladno locality, Kladno-Rakovník Basin, Kasimovian (Lower Stephanian); No. E3589 is from the Lubná locality, Kladno-Rakovník Basin, Moscovian (Bolsovian); E1152 is from the Mirošov locality, Kladno-Rakovnk Basin, Moscovian (Asturian); No. E1148 is from the Doubrava locality, Kladno-Rakovník Basin, Moscovian (Asturian); No. E1147 is from the Kladno locality, Kladno-Rakovník Basin, Moscovian (Bolsovian).

A description of the in situ spores is as follows: The trilete spores are circular, subtriangular, or oval in amb. The laevigate exine of the microspores is up to 2 µm thick. The rays of the trilete mark reach a third to two-thirds of the radius. Secondary folds of the exine possess variable sizes, shapes, positions, and numbers (Figure 15q and Figure 16c,d). The size range is 30 (61) 110 µm in diameter (Table 10). The labrum is 2–4 µm broad and is sometimes developed. Some specimens have irregular fragments of perisporial tissue (Figure 15o,p,r and Figure 16a–d). The in situ microspores can be compared to the dispersed miospore species *Calamospora microrugosa*, *C.* cf. *pedata*, *C. straminea*, and *C. breviradiata.*

*Calamostachys* cf. *ramosa* Weiss 1884

Figure 17a–l

Material: No. E3634 is from the Maria-Julia Mine, Žacléř, Intra-Sudetic Basin, Bashkirian (Langsettian-Duckmantian); No. E3627 is from the Ronna Mine, Kladno-Rakovník Basin. Moscovian (Bolsovian).

**Figure 17:**
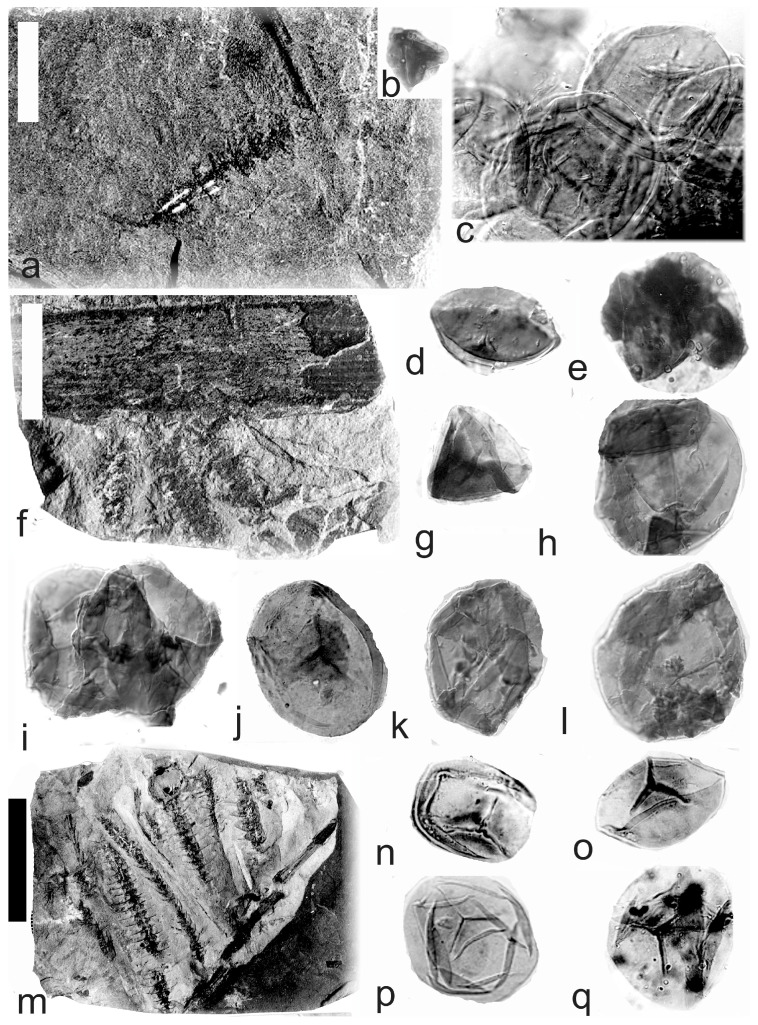
(**a**–**l**) *Calamostachys* cf. *ramosa* Weiss, specimen E3627, Ronna Mine, Kladno-Rakovník Basin, Moscovian. (**a**) General view of specimens. Scale bar 15 mm. (**b**) Immature in situ microspore. ×500. (**c**) In situ microspores of the *Calamospora* type. ×500. (**d**) In situ microspore of the *Calamospora* type. Note one major fold covering the body. ×500. (**e**) In situ microspore of the *Calamospora* type with fragments of probable tapetal tissues. ×500. (**f**–**l**) Specimen E E3634, Maria-Julia Mine, Žacléř, Intra-Sudetic Basin. (**f**) General view of the specimen. Scale bar 15 mm. (**g**) Immature in situ microspore. ×500. (**h**–**l**) In situ microspores of the *Calamospora* type. Note the fragments of probable tapetal tissues. (**j**) Note the dark contact area. All ×500. (**m**–**q**) *Calamostachys intermedia* Feistmantel. Specimen E2410, Stradonice locality near Beroun, Kladno-Rakovník Basin. (**m**) General view of the specimen. Scale bar 40 mm. (**n**–**q**) In situ microspores of the *Calamospora* type. All ×500.

A description of the in situ spores is as follows: The trilete spores are circular, subtriangular, or oval in amb. The laevigate exine of the microspores is up to 2 µm thick. The rays of the trilete mark reach a third of the radius. Secondary folds of the exine possess variable sizes, shapes, positions, and numbers (Figure 17c,d,h,j,l). The size range is 48 (62) 89 µm in diameter. The in situ microspores can be compared to the dispersed miospore species *Calamospora* cf. *microrugosa*. Some specimens are 49 (65) 99 µm in size and are enveloped by a thin monopseudosaccate layer and can resemble some forms of the miospore genera *Auroraspora*, *Remysporites*, *Perotrilites*, *Phyllothecotriletes*, or even *Diaphanospora,* and some others have irregular fragments of perisporial tissue (Figure 17e,h,i,k,l).

*Calamostachys intermedia Feistmantel 1872*

Figure 17m–q

Material: Nos. E2410 and E1174 are from the Stradonice locality near Beroun, Kladno-Rakovník Basin, Moscovian (Bolsovian).

A description of the in situ spores is as follows: The trilete spores are circular, subtriangular, or oval in amb. The laevigate exine of the microspores is up to 2 µm thick. The rays of the trilete mark reach half to two-thirds of the radius. Secondary folds of the exine possess variable sizes, shapes, positions, and numbers (Figure 17n–q). The size range is 34 (85) 137 µm. The labrum is 2–4 µm broad and is sometimes developed (Figure 17n,o). The in situ microspores can be compared to the dispersed miospore species *Calamospora* cf. *liquida* and *C.* cf. *pedata.*

*Calamostachys grandis* (Zeiller) Jongmans 1911

Figure 18a–f

Material: Collection of S. Opluštil, No. 1625, from the Kladno locality, Kladno-Rakovník Basin, Moscovian (Bolsovian).

**Figure 18:**
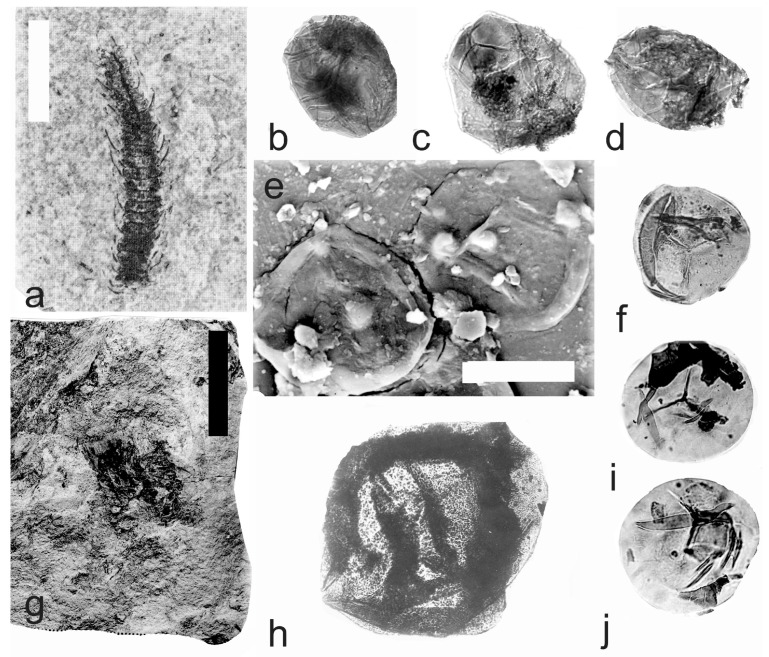
(**a**–**f**) *Calamostachys grandis* (Zeiller) Jongmans, specimen 1625, Kladno locality, Kladno-Rakovník Basin, Moscovian. (**a**) General view of the specimen. Scale bar 12 mm. (**b**–**d**) In situ microspores of the *Calamospora* type. Note the fragments of probable tapetal tissues. All ×500. (**e**) In situ microspores of the *Calamospora* type. SEM, scale bar 30 µm. (**f**) In situ microspore of the *Calamospora* type. ×500. (**g**–j) *Calamostachys* sp. E3639, Tuchlovice locality, Kladno-Rakovník Basin, Moscovian. (**g**) General view of the specimen. Scale bar 25 mm. (**h**) In situ megaspore of the *Calamospora* type. ×150. (**i**,**j**) In situ microspores of the *Calamospora* type. All ×500. *Calamostachys* sp.

A description of the in situ spores is as follows: The trilete spores are circular, subtriangular, or oval in amb. The laevigate exine of the microspores is up to 2 µm thick. The rays of the trilete mark reach a third to a half of the radius. Secondary folds of the exine possess variable sizes, shapes, positions, and numbers (Figure 18b–f). The size range is 52 (58) 70 µm. The in situ microspores can be compared to the dispersed miospore species *Calamospora breviradiata.*

Figure 18g–j

Material: Nos. E3638 and E3639 are from the Tuchlovice locality, Kladno-Rakovník Basin, Moscovian (Bolsovian).

A description of the in situ spores is as follows: The trilete spores are circular, subtriangular, or oval in amb. The laevigate exine of the microspores is up to 2 µm thick. The rays of the trilete mark reach three-quarters of the radius. Secondary folds of the exine possess variable sizes, shapes, positions, and numbers (Figure 18i,j). The size range of the microspores is 45 (67) 82 µm, and the megaspores are (Figure 18h) 610 (703) 815 µm. The labrum is 2–4 µm broad and is sometimes developed. The in situ microspores can be compared to the dispersed miospore species *Calamospora* cf. *liquida* and *C.* cf. *flexilis*, and the megaspores are classified as *Calamospora* sp. due to their large diameters.

*Huttonia* Sternberg 1837

*Huttonia spicata* Sternberg 1837

Figure 19

Material: No. E3614 is from the Ovčín locality, Radnice Basin, Moscovian (Bolsovian); Nos. E2419, E75, and E76 are from the Vranovice locality, Kladno-Rakovník Basin, Moscovian (Bolsovian).

**Figure 19:**
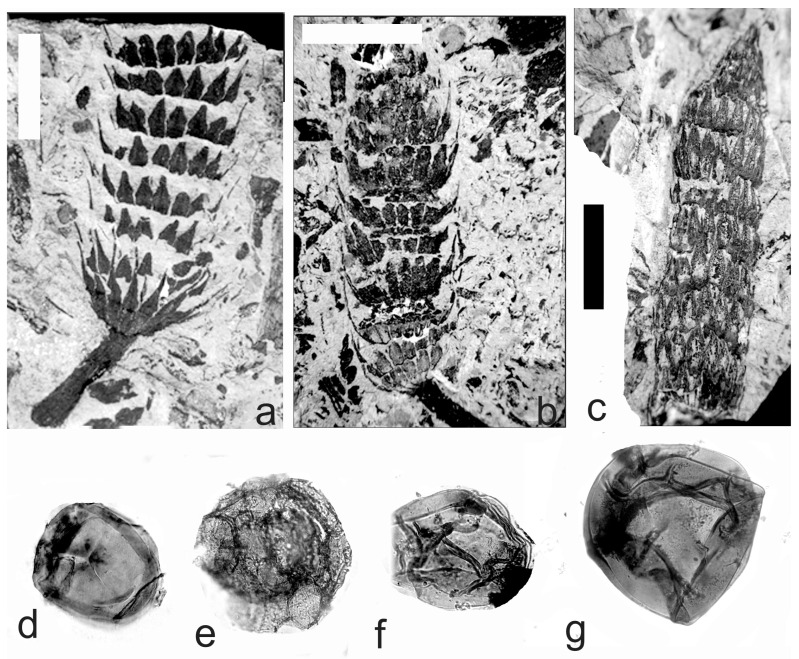
*Huttonia spicata* Sternberg. (**a**) Specimen E74, Vranovice locality. Radnice Basin, Moscovian, Scale bar 20 mm. (**b**) Specimen E2419. Vranovice locality. Radnice Basin, Moscovian. Scale bar 20 mm. (**c**) Specimen E75. Vranovice locality. Radnice Basin, Moscovian. Scale bar 20 mm. (**d**) In situ microspore of the *Calamospora* type. ×500. (**e**) In situ microspores of the *Calamospora* type. Note the outer exine monopseudosaccate-like layer. ×500. (**f**) In situ microspore of the *Calamospora* type. ×500. (**g**) In situ megaspore of the *Calamospora* type. ×120.

A description of the in situ spores is as follows: The trilete spores are circular, subtriangular, or oval in amb. The laevigate exine of the microspores is up to 1–4 µm thick. The rays of the trilete mark reach a third to two-thirds of the radius. Secondary folds of the exine possess variable sizes, shapes, positions, and numbers (Figure 19d,f,g). The size range of the microspores is 66 (84) 114 µm, and the megaspores are (Figure 19g) 115 (166) 240 µm. The labrum is 2–4 µm broad and is sometimes developed. The in situ microspores can be compared to the dispersed miospore species *Calamospora* cf. *breviradiata*, *C.* cf. *pedata*, and the megaspores are of the *Calamospora laevigata* type. Some specimens are enveloped in a thin monopseudosacccate exine layer (Figure 18e) and can resemble some forms of miospore genera *Auroraspora*, *Remysporites*, *Perotrilites*, or even *Diaphanospora*.

## 5. *Calamospora* Spores

### 5.1. Morphological Criteria

The majority of morphological features are important for the classification of dispersed calamospores, but some others, like the thickness of the exine and the sculpture, are not as significant. Some species are morphologically closely similar, and it is possible that they can be synonymous. We can divide the dispersed calamospores into a few main morphological groups, e.g., by contact area, by contact area and labrum, and only by the labrum.

Only a few authors have described a significant number of calamitean cones and their in situ *Calamospora* micro- and megaspores [11,15,24].

#### 5.1.1. Diameter

The size range given for every dispersed *Calamospora* species is not a significant criterion for their natural classification. In every *Calamospora* in situ population, the size range is variable, with specimens ranging from about 20–30 µm to more than 100 µm. One of the morphological species groups shares the same features and may be the only criterion for distinguishing it among different species of dispersed spore species [11]. The average size range of the in situ *Calamospora* microspores is 24 (75.02) 181 µm, and 147.0 (399.15) 815.0 µm for the *Calamospora* megaspores. The average difference (i.e., among the smallest and largest spores in a slide) in size is 40 µm. Dispersed calamospores are assigned to miospore and megaspore species based on their size, but the arbitrary size criterion of at least 200 µm for megaspores need not always correspond with their biological function. For example, the spore population from *Palaeostachya elongata* (E3631) is divided into two size groups. The smaller group ranges in size from 35 to 55 µm, and the larger group ranges from 105 to 150 µm in diameter. Similarly, in another specimen of *P. elongata* (E1121), spores fall into two size categories: the first category includes spores 35 to 55 μm in diameter, and the second group includes spores ranging from 85 to 140 μm in diameter. The definition of 200 μm or more for megaspores is artificial and may not apply to many *Calamospora* megaspores. The diameter of calamospores can be influenced by stages of maturity. It seems that calamitean cones matured gradually from apical to basal sporangia. The difference in the diameter for *Calamospora* from apical and basal sporangia is 5 (12) 16 µm, with an extreme example of cones of *Palaeostachya elongata*, where it is 79 µm. The difference between relatively immature and mature specimens was about 91 µm on average for the megaspores.

#### 5.1.2. Contact Area

Usually, all spores in a *Calamospora* in situ population either do or do not have a contact area. This could mean that the occurrence of the contact area may be a reliable criterion for the classification of dispersed calamospores. However, the number of in situ microspores with a contact area is higher in apical than in basal microsporangia, i.e., it implies that this morphological feature may be related to different degrees of maturity because the contact area means that the exine at the proximal pole is thickened and the thickness of the exine may be influenced by stages of maturity. The diameter of the contact area is usually equal to the length of the rays of the trilete marks or maybe sometimes slightly shorter. Sometimes, under SEM, the contact area can be seen to be slightly elevated, i.e., it is slightly thickened proximally. We know (minimally) of ten Pennsylvanian *Calamospora* species with contact areas with a size range from 34 to 146 µm and with the length of the trilete marks being a third to two-thirds of the radius. This indicates that the size of the contact area is not an easy way to distinguish among *Calamospora* dispersed species.

#### 5.1.3. Length of Rays of the Trilete Mark

The length of the rays of the trilete mark of a majority of *Calamospora* specimens is from a third to three-fourths of the radius. Almost all dispersed calamospores fit into this size range. Only a few dispersed *Calamospora* species have longer rays of the trilete mark, e.g., *Calamospora liquida*.

#### 5.1.4. Labrum

The presence or absence of a labrum need not be a constant morphological feature. Some spores from one sporangium possess a labrum, but some other spores from the same sporangium do not. A labrum is usually seen well using SEM. Only a few dispersed *Calamospora* species are defined with a labrum, e.g., *C. flava* and *C. elliptica*.

#### 5.1.5. Secondary Folds of Exine

We can imagine *Calamospora* spores in sporangium like small circular balloons without any folding, only with trilete marks on the exine surface. Due to the fossilization (adpression specimens, not petrifactions), the spore body is compressed, and the thin laevigate exine becomes folded. Different types of folding are purely occasional and not biological. Many dispersed *Calamospora* species are distinguished based on different foldings. It shows the arbitrary character of the classification of dispersed *Calamospora* species. For example, *C. pedata* is typified by one major secondary fold that covers about a half of the spore body; *C. flexilis* is typical by its folds parallel with the rays of the trilete mark, and *C. mutabilis*, *C. straminea*, *C. parva*, and *C. breviradiata* have folds parallel with the margins of the spores.

#### 5.1.6. Fragments of Tapetal Tissues

Sometimes, it is possible to observe calamospores with various fragments of probable tapetal tissues on the exine surface. These fragments have irregular sizes, shapes, thicknesss, and numbers. Sometimes, they cover the majority of the exine surface, and sometimes, only a small part. These forms are not comparable to any dispersed miospore species and are more likely related to the ontogenetic stages.

Another feature associated with the ontogenetic stages is a delicate circular monopseudosaccate-like layer that envelopes spores of the *Calamospora* type. These forms were described in [25] for the *Calamostachys calathifera* [26], *C. williamsoniana* [27], *Palaeostachya feistmanteli*, *Calamostachys calathifera*, *C. binneyana*, *C. germanica* [28,29], *C. incrassata*, and *Huttonia spicata*. Here, these forms were observed in spore populations isolated from *Palaeostachya feistmanteli*, *P. distachya*, *Calamostachys longibracteata*, *C. carinata* and *C.* cf. *ramosa*, and *Huttonia spicata*. Some authors [26,27] compared these forms with some species of miospore genera, such as *Auroraspora*, *Perotrilites*, *Remysporites*, *Callialasporites*, *Phyllothecotriletes*, or even *Diaphanospora*. But they are immature forms, i.e., younger ontogenetic stages, and are not different spore taxa. Fully matured spores lack a monopseudosaccus-like layer.

## 6. Elaterites

Spores of the genus *Elaterites* Wilson are very rare in the dispersed record. The genus was established [30] for spores with three elaters enveloping a central body of the *Calamospora* type. These microspores are very rarely reported [30,31,32,33]. Seventeen species of genera, such as the *Calamocarpon* Baxter, *Calamostachys*, *Mazostachys*, *Palaeostachya*, *Pendulostachys* Good, *Pothocites* Paterson, and *Weissistachys* Rothwell & Taylor, yielded spores of the *Elaterites* type from mainly petrified (Table 11) [9,21] and adpression [15] specimens. The size range of these microspores is 38 (78) 280 µm, and almost all of them are 38–112 µm in diameter, except for those isolated from *Calamostachys americana* Arnold, which is unusually large (140–280 µm) [10]. Some authors [10,12,34] propose that all calamiteans produced microspores of the *Elaterites* type. We have studied hundreds of palynological slides with in situ *Calamospora* populations in different stages of ontogeny, macerated from sixty specimens of calamitean cones, and we have never seen any elaters or any elater-like structures or their fragments. It is evident that there is a group of calamitean cones that produced spores of the *Elaterites* type, but the majority of them yielded only spores of the *Calamospora* type. Some authors [10,12,24] have proposed that *Vestispora* represents an ontogenetic stage of *Calamospora*. The authors of [35] excluded this theory based on some main points, and the authors confirmed their conclusions.

*Vestispora* is characterized by a homogeneous outermost exine layer and small circular operculum lying above the proximal pole of the central body of the *Calamospora* type. *Elaterite* spores lack an operculum, and there is always a space among three elaters, i.e., the outer layer is not homogeneous. Elaters of *Elaterites* originate from a small triangular area on the distal pole, i.e., opposite to the proximal surface with a small circular operculum. Anything resembling such a triangular distal structure has never been observed on the *Calamospora* spores described herein. The structure of *Elaterites* is monotonous, but the sculpture of *Vestispora* is variable, including laevigate, foveolate, costate, and primary and secondary reticulate.

The second point is that *Vestispora* and *Calamospora* spores were produced by different plants. *Vestispora* was not produced by any calamiteans. The major *Vestispora* producer was one group of sphenophyllaleans [33]. Another small group of sphenophyllaleans produced calamospores but only *Calamospora* and not *Calamospora* and *Vestispora* together. *Calamospora* ranges from the Devonian to the Cenozoic, whereas *Vestispora*-producers range from the Brigantian to late Kasimovian. As a consequence, almost all *Vestispora*-producing sphenophyllaleans are good stratigraphical markers, but calamitean spores are not. *Elaterites* have an even shorter stratigraphical range, ranging through only a part of the Pennsylvanian [36]. Almost all *Vestispora*-producing sphenophyllaleans are good stratigraphical markers [37], but calamiteans are not. *Elaterites* have an even shorter stratigraphical range, only a part of the Pennsylvanian [35].

The third point concerns different ultrastructure sections of the exine (TEM) of *Elaterites* [38] and *Vestispora* [39]. Elaters of *Elaterites* are three-layered [39], while the exospore of *Vestispora* is only bi-layered [40].

When in situ, *Elaterites* are reported only from coal-ball calamitean cones and not from adpressions. The only roughly similar spore structures are hygroscopic elaters of the recent *Equisetum* spores, which show that their ultrastructure is different. The sporoderm of *Equisetum* spores has an endospore, exospore, perispore, and bi-layered elaters [38], but the *Elaterites* are only bi-layered, and elaters have three layers.

*Vestispora* spores have a circular laevigate central body with trilete marks and outer exospores with a circular operculum. Sometimes, in palynological slides with in situ *Vestispora,* it is possible to observe various degrees of mechanical damage in the exospores and that the central body is of the *Calamospora* type. However, *Vestispora* is not a relatively immature *Calamospora* because we have never observed any ontogenetic stages of *Vestispora.* Table 11 shows all the *Elaterites* producing calamitean plants.

## 7. Parent Plants

The main *Calamospora* producers are calamitean cones, especially the genera *Calamostachys*, *Palaeostachya*, and *Macrostachya.* Minor producers of *Calamospora* are the genera Pothocites, *Paracalamostachys* Weiss, *Huttonia*, *Weissistachys*, *Cingularia* Weiss, *Pendulostachys*, and *Calamocarpon*. Non-calamitean producers are sphenophyllaleans (Table 12) and noeggerathialeans. Sphenophyllaleans and calamiteans are closely related. The noeggerathialean genus *Discinites* K. Feistmantel produced micro- and megaspores of the *Calamospora* type and *Noeggerathiaestrobus* O. Feistmantel-only *Calamospora* megaspores (Table 12).

*Calamospora* was produced by several parent plant species of different affinity [7], but its main producers were Pennsylvanian calamiteans. The first record of the in situ spores was interpreted as *Calamospora* (*Calamospora atava* (Naumova) McGregor and *C. pannucea* Richardson) or *Retusotriletes* type) and is known as the zosterophyll species *Sawdonia acanthotheca* Gensel et al. [41]. Note that *Retusotriletes* is curvaturate and, therefore, distinct from *Calamospora* and that most of the zosterophylls possess *Retusotriletes*-type spores. *Calamospora*-type spores, which are reported in some zosterophylls, such as *S. acanthotheca,* may be immature. From the Paleozoic plants of possible lycophyte affinity, we know only one record of an in situ *Calamospora*, Mississippian genus *Eleutherophyllum* Stur [42].

Other non-calamitean *Calamospora* records [7] include the Triassic *Bustia ludowici* Grauvogel-Stamm, *Echinostachys cylindrica* Schimper and Mougeot, *E. oblongus* Brongniart (*Calamospora keuperiana* and *C. mesozoica* types), *E. verticillata* Grauvogel-Stamm (*Calamospora tener* type), *Equisetostachys nathorstii* Halle, *E. suecius* (Nathorst) Halle (*Calamospora mesozoica* type), and Devonian *Protobarinophyton obrutschevii* Ananiev (*Calamospora atava* type.

## 8. Conclusions

A comparison of hundreds of palynological slides with in situ populations, isolated from sixty-one specimens of Pennsylvanian calamitean cones belonging to sixteen species of four genera, allows the definition of morphological variations of in situ *Calamospora*. The classification of dispersed *Calamospora* is purely arbitrary because all morphological criteria, including the diameter, thickness of exine, number, shape, position, and size of the secondary folds of the exine, as well as the length of rays of the trilete marks, are variable within one in situ *Calamospora* population. Some features are variable, and others are related to different ontogenetic stages.

The calamitean cones matured gradually from the base to the apex, as demonstrated by the different diameters of spores and a more frequent occurrence of contact areas on the spores isolated from basal and apical sporangia. The calamitean cones were bisporangiate, and an arbitrary size criterion for the division of micro- and megaspores (200 µm) may not be biologically meaningful. Some cones yielded spores with a bimodal size distribution, so even though the larger spores were less than 200 μm, they probably represent megaspores because the size in these populations exhibits a bimodal curve.

There is no evidence that *Elaterites* and *Vestispora* are ontogenetic stages of *Calamospora*, as demonstrated by the morphological and stratigraphical differences. However, it is evident that some Paleozoic calamitaleans produced not only *Calamospora* but also the *Elaterites* spores.

The majority of Paleozoic *Calamospora* producers were calamitean cones, mainly the genera *Calamostachys*, *Palaeostachya*, and *Macrostachya*. Sphenophyllaleans were a minor source of *Calamospora*. Some noeggerathialeans also produced *Calamospora*.

## Figures and Tables

**Table 1:** Stratigraphical position of studied calamitean cones.

Period	Age	
303 Ma	Kasimovian	Stephanian
Pennsylvanian 307 Ma	Moscovian	Asturian
		Bolsovian
315 Ma	Bashkirian	Duckmantian
		Langsettian

**Table 2:** The size of the in situ spores of *Palaeostachya ettingshausenii* Kidston.

Specimen Number	Diameter of In Situ Spores (µm)
E 2500—basal part	55.0 (67.64) 84.0
apical part	44.0 (62.3) 72.0
E 2412 right cone, basal part	84.0 (121.1) 181.0
apical part	62.0 (105.77) 131.0
left cones	461.0 (576.75) 708.0
E 3623	56.0 (90.27) 121.0
E 3625	58.0 (92.33) 126.0
E 2414	48.0 (71.19) 101.0
E 1117 upper cone, basal part	49.0 (64.6) 74.0
middle part	48.0 (61.22) 79.0
lower cone	43.0 (63.2) 74.0
E 3596	56.0 (92.25) 131.0
E 3622	76.0 (84.52) 94.0
	234.0 (398.58) 602.0
E 3624	390.0 (494.38) 621.0
E 3618	330.0 (420.88) 592.0 54.0 (69.99) 79.0
E 3626	75.0 (119.5) 151.0
935 left cone, basal part	60.0 (83.1) 100.0
apical part	52.0 (67.44) 74.0
right cone, basal part	71.0 (75.97) 80.0
apical part	58.0 (64.22) 70.0
1451 left cone, basal part	64.0 (71.63) 89.0
right cone, basal part	60.0 (67.81) 80.0
apical part	52.0 (59.32) 70.0

**Table 3:** Size of the in situ spores of *Paleostachya distachya*.

Specimen Number	Diameter of In Situ Spores (µm)
E 3599	54.0 (64.36) 79.0
E 3603	55.0 (63.14) 76.0
E 3500	55.0 (66.2) 78.0
E 3608	36.0 (60.82) 108.0
E 3598	41.0 (62.6) 99.0
E 1132	33.0 (76.4) 92.0
E 1134	59.0 (90.58) 119.0
E 3604	71.0 (87.38) 111.0

**Table 4:** Size of the in situ spores of *Palaeostachya elongata*.

Specimen Number	Diameter of In Situ Spores (µm)
E 3607	84.0 (112.2) 123.0
E 3631	35.0 (42.35) 50.0, 108.0 (123.35) 149.0
E 3616	84.0 (124.9) 152.0
E 1122	49.0 (75.93) 92.0
E 1121	35.0 (44.5) 56.0, 82.0 (103.42) 141.0

**Table 5:** Size of the in situ spores of *Palaeostachya gracillima* Weiss.

Specimen Number	Diameter of In Situ Spores (µm)
E 3635	48.0 (65.94) 83.0
E 1127	24.0 (62.57) 83.0
E 1119	42.0 (68.38) 95.0

**Table 6:** Size of the in situ spores of *Macrostachya carinata* (Germar) Zeiller.

Specimens Number	Diameter of In Situ Spores (µm)
E 3541	66.0 (81.9) 102.0
E 3637 basal part	265.0 (318.57) 366.0
E 3637 middle part	221.0 (294.47) 372.0
E 3637 apical part	156.0 (203.2) 232.0
E 3637 apical part	58.0 (64.11) 69.0
E 1178	69.0 (119.67) 167.0
E 1180	183.0 (244.8) 342.0
E 1181	71.0 (93.0) 116.0

**Table 7:** Diameters of the in situ megaspores of the *Calamospora* type isolated from different portions (basal, middle, and apical) of the cone of *Macrostachya carinata* (Germar) Zeiller.

Number of Specimens	Diameter of Megaspores (µm)
E 3637 basal part	265.0 (318.57) 366.0
E 3637 middle part	221.0 (294.47) 372.0
E 3637 apical part	156.0 (203.2) 232.0

**Table 8:** Size of the in situ spores of *Calamostachys germanica* Weiss.

Specimen Number	Diameter of In Situ Spores (µm)
E 3620	75.0 (107.4) 126.0, 181.0 (247.83) 329.0
E 3621	261.0 (389.5) 485.0
E 2408	54.0 (61.27) 73.0
E 5641	55.0 (62.43) 91.0
E 1159	54.0 (79.9) 96.0
E 1161	180.0 (350.56) 438.0
E 2409	147.0 (442.05) 684.0

**Table 9:** Size of the in situ spores of *Calamostachys longibracteata* Němejc.

Specimen Number	Diameter of In Situ Spores (µm)
E 3605	45.0 (54.5) 72.0
E 1154	45.0 (63.33) 78.0
E 1155	48.0 (58.5) 77.0
E 1163	47.0 (62.21) 84.0

**Table 10:** Size of the in situ spores of *Calamostachys tuberculata* (Sternberg) Jongmans.

Specimen Number	Diameter of In Situ Spores (µm)
E 3589	318.0 (352.67) 396.0
E 1147 basal part	56.0 (67.36) 74.0
apical part	54.0 (64.3) 75.0
E 1148	51.0 (65.21) 78.0
E 1152	52.0 (65.9) 79.0
1238	31.0 (66.23) 110.0
1951	30.0 (62.6) 107.0

**Table 11:** Parent plants produced spores of the Elaterites type.

Parent Plant	Diameter of Microspores (µm)	Classification of In Situ Spores	References
*Calamocarpon insigne*	38–60	*Calamospora*/*Elaterites*	[24] Good 1975
*Calamostachys americana*	140–280	*Calamospora*/*Elaterites*	[24] Good 1975
*C. binneyana*	38–61	*Calamospora*/*Elaterites*	[24] Good 1975
*C. casheana*	75	*Calamospora*/*Elaterites*	[24] Good 1975
*C. inversibractus*	44–74	*Calamospora*/*Elaterites*	[24] Good 1975
*C. ludwigi*	74–112	*Calamospora*/*Elaterites*?	[15] Hartung 1933
*C. magnae-crucis*	45	*Calamospora*/*Elaterites*?	[7] Balme 1995
*Mazostachys noei*	45–50	*Calamospora*/*Elaterites*	[24] Good 1975
*M. pedunculata*	52–100	*Calamospora*/*Elaterites*	[24] Good 1975
*Palaeostachya andrewsii*	56–100	*Calamospora*/*Elaterites*	[24] Good 1975
*P. decacnema*	54	*Calamospora*/*Elaterites*	[24] Good 1975
*P. distachya*	70–105	*Calamospora*/*Elaterites*?	[24] Good 1975
*P. feistmanteli*	45–95	*Calamospora*/*Elaterites*?	[26] Serret Brousmiche 1986
*P. vera*	75–80	*Calamospora*/*Elaterites*	[24] Good 1975
*Pendulostachys cingulariformis*	63–89	*Calamospora*/*Elaterites*	[24] Good 1975
*Pothocites grantoni*	82–104	*Calamospora*/*Elaterites*?	[7] Balme 1995
*Weissistachys kentuckiensis*	39–75	*Calamospora*/*Elaterites*	[24] Good 1975

**Table 12:** Paleozoic spores of Equisetales and their parent plants.

Spore Genus	Calamiteans
*Calamospora* (micro- and megaspores)	*Pothocites grantonii*, *P. pettycurensis*, *Calamostachys americana*, *C. Binneyana*, *C. calathifera*, *C. casheana*, *C. dumasi*, *C. germanica*, *C. grandis*, *C. gunlongii*, *C. incrassata*, *C. intermedia*, *C. inversibractus*, *C. longibraceata*, *C. ludwigi*, *C. magnae-crucis*, *C. paeniculata*, *C.* cf. *ramosa*, *C. solmsii*, *C. tuberculata*, *C.* cf. *tuberculata*, *C. williamsoniana*, *C. zeilleri*, *C*. sp (*sensu* Moore), *C.* sp. A (*sensu* Bek), *C.* sp. B (*sensu* Serret and Brousmiche), *Macrostachya carinata*, *M. carinata* var. *approximata*, *M. caudata*, *M. hauchecornei*, *M. infundibuliformis*, *M. thompsonii*, *Palaeostachya andrewsii*, *P. aperta*, *P. decacnema*, *P. dircei*, *P. distachya*, *P. elongata*, *P. ettingshausenii*, *P. equisetoformis*, *P. feistmantelii*, *P. gracilis*, *P. gracillima*, *P. pedunculata*, *P. superba*, *P. thuringiaca*, *P. trabeculata*, *P. vera*, *P* sp. (*sensu* Moore), *P.* sp. A (*sensu* Bek), *Paracalamostachys scatervillei*, *P. heterospora*, *P. minor*, *P. spadiciformis*, *P. striata*, *Macrostachya noei*, *M. pendulata*, *Kallostachys scottii*, *Huttonia spicata*, *Weissistachys kentuckiensis*, *Cingularia typica*, *Calamocarpon insignis*, *Pendulostachya cinguliformis*
*Elaterites*	*Calamostachya americana*, *C. inversibractus*, *Palaeostachya andrewsii*, *P. decacnema*, *Macrostachya noei*, *M. pendulata*, *Weissistachys kentuckiensis*, *Calamocarpon insignis*, and *Pendulostachys cinguliformis*
	Sphenophyllaleans
*Calamospora*	*Bowmanites moorei*, *B. myriophyllus*, *B. nindelii*, *B. priveticensis*, *B. stimulosus*, *B. verticillatus*, *B.* sp. *(herein)*, *B. tenerrimum*, *Sphenophyllum aguensis*, *S. beinertii*, *S. waldenburgense*, *Cheirostrobus pettycurensis*, *Pothocites grantonii*, and *P. pettycurensis*
	Noeggerathialeans
*Calamospora* (micro- and megaspores)	*Discinites bohemicus*, *D*. sp. (cf. *bohemicus*), *D. delectus*, *D. hanchengensis*, *D. hlizae*, *D. major*, *D. nemejcii*, *D.* cf. *raconicensis*, *D. vicinalis*, *Lacoea seriata*, *Paratingia wudensis*, *Tingia unita*, *T* sp., *Tingiostachya tetralocularis*, and *T.* sp., *Noeggerathiaestrobus* (only megaspores)
*Verrucosisporites*	*Noeggerathiaestrobus bohemicus*

## Data Availability

Material including plant specimens is stored in the National Museum, Prague, Czech Republic and palynological slides are in the Institute of Geology, Academy of Sciences of the Czech Republic, Prague, Czech Republic.
